# Spatial transcriptomics and histopathological analyses reveal potential biomarkers and immune features linked to recurrence in head and neck cancer

**DOI:** 10.3389/fimmu.2026.1713732

**Published:** 2026-08-11

**Authors:** Chameera Ekanayake Weeramange, Yibi Chen, Kym-Mai Nguyen, Xi Zhang, Gunter Hartel, Andrew Causer, Onkar Mulay, Prakrithi Pavithra, Pui Yeng Lam, Jazmina Gonzalez Cruz, Sarju Vasani, Lizbeth Kenny, Touraj Taheri, Omar Breik, Quan Nguyen, Chamindie Punyadeera

**Affiliations:** 1 Saliva and Liquid Biopsy Translational Laboratory, Institute for Biomedicine and Glycomics Griffith University, Brisbane, QLD, Australia; 2 Institute for Molecular Bioscience, The University of Queensland, Brisbane, QLD, Australia; 3 Statistics Unit, Queensland Institute of Medical Research (QIMR) Berghofer Medical Research Institute, Brisbane, QLD, Australia; 4 School of Public Health, The University of Queensland, Brisbane, QLD, Australia; 5 School of Nursing, Queensland University of Technology, Brisbane, QLD, Australia; 6 Frazer Institute, Faculty of Medicine, The University of Queensland, Brisbane, QLD, Australia; 7 Faculty of Medicine, The University of Queensland, Brisbane, QLD, Australia; 8 Department of Otolaryngology, Royal Brisbane and Women’s Hospital, Brisbane, QLD, Australia; 9 Department of Cancer Care Services, Royal Brisbane and Women’s Hospital, Brisbane, QLD, Australia; 10 Department of Anatomical Pathology, Royal Brisbane and Women’s Hospital, Brisbane, QLD, Australia; 11 Department of Oral and Maxillofacial Surgery, Royal Brisbane and Women’s Hospital, Brisbane, QLD, Australia

**Keywords:** biomarkers, head and neck cancer, spatial transcriptomics, tumor microenvironment, tumor recurrence

## Abstract

**Introduction:**

Head and neck cancers (HNCs) are aggressive malignancies, with recurrence contributing substantially to poor patient outcomes. We used spatial transcriptomics to identify tumour and immune features associated with recurrence.

**Methods:**

Pathologist-annotated tumour and stromal regions from recurrent and non-recurrent HNC samples were analysed using spatially resolved transcriptomics. Exploratory differential expression analysis was used to nominate tumour-associated candidate markers for recurrence. Selected candidates were assessed by immunohistochemistry and further evaluated using independent spatial transcriptomic and bulk RNA-sequencing cohorts. Immune-cell abundance and spatial co-localisation were also examined.

**Results:**

*PAEP*, *MARCO*, *PNPLA3*, and *FMO2* emerged as leading candidate markers associated with recurrence. Increased expression of PAEP, MARCO, and PNPLA3 was supported by immunohistochemistry. Integrative analysis across independent spatial and bulk RNA cohorts demonstrated consistent PAEP overexpression in recurrent HNC, particularly within tumour regions, with this pattern reproduced across datasets and profiling platforms. Non-recurrent tumours showed a trend towards greater T- and B-cell infiltration, with these populations frequently co-localising within the tumour microenvironment. In recurrent tumour regions, PAEP expression showed an inverse trend with B-cell and plasma-cell abundance, which was higher in non-recurrent samples.

**Discussion:**

Spatial transcriptomics identified tumour-intrinsic and immune microenvironmental features associated with HNC recurrence. PAEP emerged as the most reproducible candidate biomarker across internal and external cohorts and may be associated with reduced B-cell and plasma-cell infiltration. These findings support further validation of PAEP and related immune signatures for recurrence-risk stratification and personalised treatment.

## 1 Introduction

Head and neck cancers (HNC) are responsible for over 900, 000 new cases and 450, 000 deaths annually, making them one of the deadliest and most debilitating cancers globally (1). Despite advancements in diagnostic and therapeutic strategies, HNC patients’ overall 5-year survival remains low, at 50-60% (2–5), with recurrence being a key driver of these outcomes.

Recurrence is known to often arise from minimal residual disease that persists after curative surgery (6–10). Additionally, tumors consist of dynamically evolving subpopulations of tumor cells that frequently acquire genetic and epigenetic changes (11, 12), underpinning plasticity that may lead to therapy resistance and tumor recurrence. Similarly, the ability of tumor cells to disseminate into secondary sites contributes to cancer relapse (13, 14). Moreover, certain metastasized cells, particularly cancer stem cells, can remain dormant for extended periods before germinating at the new site, escaping treatment that targets active tumor cells (15).

Tumor microenvironment (TME) plays a pivotal role in recurrence. Immune infiltration, such as by CD8+ T lymphocytes, is associated with improved outcomes (16–18), while cancer-associated fibroblasts have been shown to promote the invasion and migration of cancer cells and hinder immune cell activity (19–21). Recent single-cell RNA sequencing (scRNA-seq) studies have illuminated the dynamic evolution of tumor and stromal compartments during HNC progression. One investigation delineated malignant epithelial trajectories spanning precancerous to recurrent disease, uncovering a tumorigenic subcluster regulated by TFDP1, alongside POSTN^+^ fibroblasts and SPP1^+^ macrophages that remodel the TME to drive progression (22). In addition, exhausted CXCL13^+^ CD8^+^ T cells were identified as facilitators of extranodal expansion during recurrence. Collectively, these findings underscore how intratumoral heterogeneity and immune dysfunction promote relapse. Technologies that can reveal interactions between cancer, immune, and stromal cells within TME, such as spatial transcriptomics, may lead to the discovery of predictive biomarkers of recurrence.

Current prognosis parameters include tumor stages, anatomical sites, HPV/p16 status, comorbidities, and smoking or alcohol history. For example, the presence of p16 as a marker for HPV infection indicates a better prognosis, longer survival, and response to HPV-driven carcinogenesis (23). While these factors contribute to stratifying risk, they do not capture biological complexity underlying recurrence. Early or regional recurrence increases the risk of death, yet molecular markers reflecting the progression at early stages remain lacking, leading to underestimation of survival variability among patients.

In this study, we aimed to discover biomarkers and characterize the TMEs of recurrent HNC by applying Visium Spatial Transcriptomics (ST) to compare recurrent and non-recurrent HNC, unbiasedly exploring spatially resolved expression of 18, 000 genes within the preserved tissue context. By integrating spatial data with histopathological assessment of cancer cores, tumor edges, and stromal regions, we identified potential biomarkers of recurrence. We assessed these candidates at the protein level and in external bulk and spatial transcriptomic datasets to identify potential mechanisms associated with recurrence in HNC. This work offers a valuable resource reporting spatially resolved expressions for advancing our understanding of tumor biology and improving prognostic modelling.

## 2 Materials and methods

**Figure 1:**
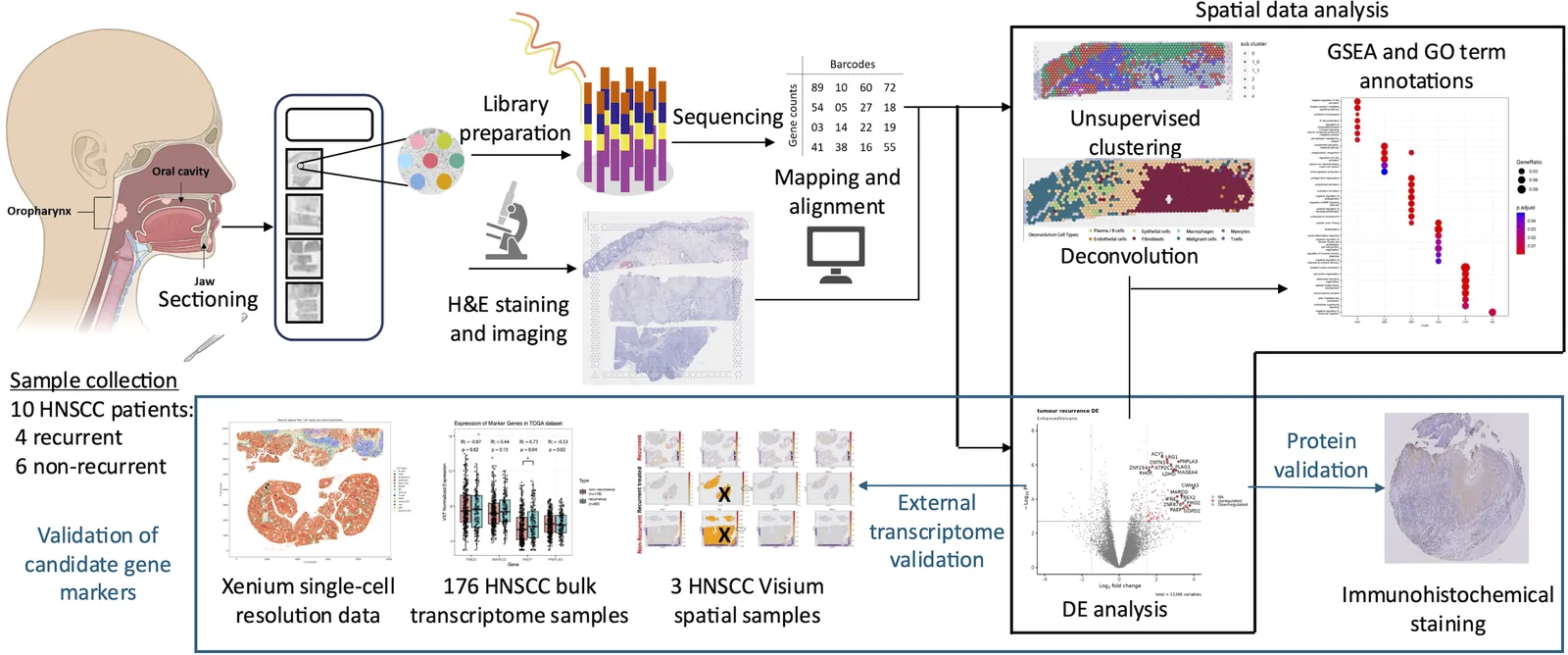
The workflow for identifying candidate genes that are associated with recurrences in HNC leveraging 10x Visium Spatial Transcriptomics technology coupled with immunohistochemical staining. The study cohort comprises a total of 10 baseline formalin fixed paraffin embedded (FFPE) tissue samples, 4 from patients who later developed recurrence and 6 from patients who remained recurrence-free during the study period of 4 years. FFPE tissues (N = 10) were obtained from HNC patients with tumors originating from the jaw, oral cavity, and oropharynx.

**Figure 1** displays the overall workflow of the study.

### 2.1 Patient recruitment

HNC patients were recruited from Royal Brisbane and Women’s Hospital (RBWH), Brisbane, following the approved ethics by the Metro South Human Research Ethics Committee, Brisbane, QLD, Australia (HREC/12/QPAH/381). Written informed consent was obtained from all HNC patients before enrolment in the study.

### 2.2 Samples preparation and sequencing

Formalin-fixed paraffin-embedded (FFPE) tumor tissues from 10 HNC patients, comprising recurrent (N = 4) and non-recurrent (N = 6), were used as described in **Supplementary Table 1**. One patient (R3) had two distinct tumor biopsies embedded into separate FFPE blocks. Both blocks were sectioned individually onto the Visium array slides but treated as a single sample during subsequent computational analyses. Clinical details are outlined in **Supplementary Table 1**.

To ensure tissue suitability for spatial transcriptomic analysis, RNA quality was assessed using DV200 score, reflecting the percentage of total profiled RNA fragments longer than 200 bp. About 5–10 sections (5µm thick) were used for RNA quality assessment using the RNeasy FFPE Kit (#73504, Qiagen). The DV200 score was determined via the BioAnalyzer electrophoresis using the RNA 6000 Pico Kit (#5067-1513, Agilent), applying a threshold of DV200>30% for study inclusion (24).

Library preparation followed the Visium Spatial Gene Expression for FFPE User Guide (CG0000407, CG000408, CG000409 – 10x Genomics), with modifications described by Vo et al. (25). FFPE blocks were sectioned at 5µm by a rotary microtome (Leica RM2245 Rotary Microtome). Sections were transferred into a preheated (41 °C) water bath for 3 minutes before being placed onto the Visium Spatial Gene Expression slide. Multiplexing was employed to optimize capture area usage. The slide was then dried at 42 °C for 3 hours and stored overnight at room temperature in a parafilm-seal 50mL-Falcon tube containing silica bead desiccants.

The following day, the slide was incubated at 60 °C for 2 hours on a thermocycler (Bio-Rad C1000 Thermal Cycler) to melt paraffin wax, immediately deparaffinized in xylene (twice, for 10 minutes), and rehydrated through an ethanol gradient (100% thrice for 3 minutes; 96% twice for 3 minutes; 70% for 3 minutes; followed by a brief water immersion). Haematoxylin and eosin (H&E) staining and imaging was performed according to the Visium Spatial for FFPE, including Deparaffinization, H&E Staining, Imaging & Decrosslinking, following the Demonstrated Protocol (CG000409 - 10x Genomics). Slide images were acquired at 20× magnification using a Zeiss Axioscan Z1 Fluorescent Imager. Following imaging, tissue sections underwent decrosslinking in HCl-preconditioned TE buffer (pH 8.0) at 70 °C for 1 hour.

For expression profiling, 25 bp probe pairs targeting the whole human transcriptome (#2000449, #2000450, 10x Genomics) were hybridized to their complementary transcript targets *in situ* overnight. Following hybridization, adjacent probe pairs were ligated to generate 50 bp ligation products. Endogenous tissue mRNA was subsequently digested using an RNase mix, releasing the stable ligated probe products from the tissue. The synthetic poly(A) tails of these released probes were captured by the slide-bound, spatially barcoded oligo(dT) primers.

Slide-bound cDNA was synthesized from the captured, ligated probes and eluted into 0.2 mL PCR tubes. Each generated cDNA molecule contained a unique spatial barcode, a Unique Molecular Identifier (UMI), and the specific probe sequence. Short-read Illumina-compatible libraries were constructed using the Visium Spatial Gene Expression Reagent Kits for FFPE (CG000407, 10x Genomics). Quantitative PCR was performed to determine the optimal Sample Index PCR cycle number, and the libraries were amplified for 15 cycles. Final library molecules were purified using SPRIselect beads, and cDNA quality and concentration were verified on a Bioanalyzer. Sequencing was conducted by the IMB Sequencing Facility on an Illumina NovaSeq platform (100-cycle kit), targeting a sequencing depth of approximately 100 million reads per library.

The corresponding H&E images were subsequently annotated by an HNC-specialized anatomical pathologist.

### 2.3 Data analyses

For full reproducibility, the code detailing the following analyses is publicly available at https://github.com/BiomedicalMachineLearning/RecurrenceHN.

#### 2.3.1 Pre-processing and quality control

Raw sequencing data and high-resolution H&E slide images were processed using the 10x Genomics SpaceRanger pipeline. Transcripts were mapped to the GRCh38 human reference transcriptome to generate spatially resolved gene expression matrices aligned with their corresponding tissue histology. The 10x Genomics Loupe Browser was utilized to manually overlay metadata, including patient recurrence status and anatomical region. To establish morphological context, discrete tissue spots were assigned a dominant cell type or histological domain based on manual annotations provided by an HNC-specialized anatomical pathologist on the matching H&E images.

Downstream quality control (QC), filtering, and integration were conducted using the Seurat (v4.1.0) R package (26). To eliminate low-quality data and technical artifacts, spots capturing fewer than 100 unique genes were excluded, and genes detected in fewer than three spots across the entire dataset were removed. Additionally, any spots located outside the boundaries of the primary tissue section or lacking pathologist-guided histological annotations were discarded.

To account for differences in sequencing depth across capture areas, the filtered raw count matrices were normalized and variance-stabilized via the SCTransform framework (27). This method applies a regularized negative binomial regression model to effectively remove technical variation while preserving biological heterogeneity. To mitigate technical batch effects and ensure cross-sample comparability, a unified integrated assay was generated for downstream clustering. This was achieved using Seurat’s anchor-based integration workflow, employing Canonical Correlation Analysis (CCA) to identify shared biological states (“anchors”) across individual sample matrices. The resulting integration vectors were applied to harmonize the datasets, ensuring that downstream clustering was driven by genuine biological features rather than inter-sample technical variation.

#### 2.3.2 Unsupervised clustering

Unsupervised clustering was performed on the SCTransform normalized and integrated dataset using the Seurat FindClusters function. The top 50 principal components (PC) were used to build the shared nearest neighors (SNN). Sub-clustering was performed on cluster 1 using the *FindSubCluster* function at 0.1 resolution in Seurat to gain better annotation of that cluster. The *FindMarker* function with Wilcoxon rank sum test (26) was used to identify upregulated marker genes for each cluster. Genes with an adjusted P<0.05 were selected to perform GO term analysis to identify gene groups and/or functions associated with each cluster group were analyzed by clusterProfiler R package, with a significance threshold of Benjamini-Hochberg- (BH-) adjusted P<0.05. Results of cluster gene markers and enriched pathway were visualised using the *dotplot*, *cnetplot* and *emapplot* functions (*clusterProfiler)*, producing dot plots, Gene-Concept Network and enrichment maps respectively (28). To find enriched clusters in recurrent or non-recurrent samples, we performed a permutation test as in the scProportionTest R package (29), using the *permutation_test* function with the default parameters (n=1, 000 permutation tests). The test calculates the relative difference in abundance of the spots annotated by a cluster between the two different conditions, providing the log_2_FC and false discovery rate (FDR). The *permutation_plot* function was used to visualize the FC between recurrence status for all clusters.

#### 2.3.3 Cell type annotation at spot level by deconvolution

Visium spot deconvolution was performed using the Conditional Autoregressive Deconvolution (CARD) R package (30). This software estimates cell-type composition within spatial transcriptomics spots by integrating cell-type-specific transcriptional profiles derived from a single-cell reference dataset with a conditional autoregressive model that accounts for spatial correlation between neighboring spots. Publicly available scRNA-seq data, generated from 37 human HNC specimens, was obtained from the National Center of Biotechnology Information’s Gene Expression Omnibus (GSE181919) (22) and used as reference. This scRNA-seq dataset consisted of samples with or without HPV, from different tissue origins and from primary and metastatic patients, matching the diversity of our dataset.

Raw count data from both spatial transcriptomics and single-cell data was used to run CARD. The cell type with the highest predicted proportion was assigned as the dominant identity for each spot. CARD was also applied to impute single-cell resolution mappings from spatial transcriptomics data. This was achieved by constructing a refined spaced grid and estimating new cell locations through calculating a conditional mean established between the original and imputed cell coordinates. This CARD function was used to generate single-cell mapped spatial data for each sample.

#### 2.3.4 Differential analysis of mRNA expression data

Differential expression (DE) analysis was performed between recurrent and non-recurrent samples in regions labeled by a pathologist as tumor, stroma and stroma-tumor infiltrating lymphocytes (TIL). DE analysis was also conducted for tumor p16-positive (n=4) and p16-negative (n=4) FFPE sections separately. By focusing the DE analysis specifically on the spots in the malignant compartment, we distill the non-specific signal from other regions of the tissue. Biological samples were randomly partitioned into three distinct groups of spots to generate triplicate pseudo-samples per patient. The exceptions were samples NR1 and NR4, which were aggregated as single pseudo-samples due to their limited number of tumor spots. For each partition, transcriptomic data were averaged across all constituent spots. This approach yielded a total of 26 pseudo-bulk profiles for differential expression (DE) analysis. Differential expression analysis was then performed on these aggregated profiles using the edgeR (v3.36.0) R package (31), which is highly robust to the sparsity and variable library sizes inherent to spatial pseudo-bulk data.

A limitation of this approach is that the three pseudo-samples derived from the same patient are not independent biological replicates. Because patient-level correlation was not modelled within a mixed-effects framework, the resulting p-values are affected by an inflated effective sample size. Strict patient-level pseudo-bulk aggregation (i.e., one pseudo-bulk per patient) was not used due to insufficient degrees of freedom, which arises from the small cohort size (n = 10), in the context of the high baseline clinical and anatomical heterogeneity of the samples. Consequently, this initial analysis was used strictly as an exploratory feature-selection tool to rank candidate genes for subsequent independent validation, rather than as a definitive statistical assessment of differential expression. We also implemented a DE analysis approach taking into account patient-level variation using linear mixed-effects model as described below.

#### 2.3.5 Gene set enrichment and GO term analysis on DE genes specific to cell types

Gene set enrichment analysis (GSEA) and Gene Ontology (GO) analysis were applied to find enriched pathways among up and down regulated genes in the recurrent tumor samples. For GO analysis the list of differentially expressed genes was filtered based on a p-value (p<0.05) and log_2_fold change (log_2_FC) >1.0 to obtain biologically meaningful pathways significantly changing between recurrence and nonrecurrence. For GSEA, the log_2_FC-ranked genes were used to calculate the enrichment score, using all expressed genes in the DE analysis (N = 13, 266) as the background genes (32). Significance was determined using the BH-adjusted p-value (p<0.05) (33). The following R packages were used to perform GO analysis: org.Hs.eg.db (v3.14.0), DOSE (3.24.2) (34), enrichplot (1.18.3), clusterProfiler (v4.2.2) (35) and ReactomePA (1.42.0) (36) and fgsea (1.24.0) (37).

#### 2.3.6 Identification of candidate genes associated with tumor recurrence using DE genes

To identify top candidate genes for tumor recurrence from a large pool of differentially expressed genes, we implemented a systematic prioritization strategy leveraging both statistical thresholds and spatial expression patterns.

First, genes were initially filtered based on an effect size threshold of |log_2_FC| > 1.5. From this subset, the top 10 genes were selected for further evaluation based on a combination of fold change magnitude and statistical significance. Selecting genes with a higher log_2_FC increases the likelihood of capturing robust biological changes that translate to the protein level, while prioritizing the lowest P values minimizes the false discovery rate.

Second, we leveraged the unique spatial resolution of our data to map gene expression directly back onto the corresponding H&E tissue images. Using the *SpatialDimPlot* function from Seurat v4.1.0, we assessed whether candidate gene expression was consistently and uniformly upregulated across malignant domains in all recurrent samples. This spatial filtering step was critical to prevent single sample expression outliers from biasing our pseudo bulk statistical significance, which represents an essential safeguard given our discovery cohort size. Genes that demonstrated consistent upregulation across multiple recurrent patients, rather than localized or sample specific spikes, were prioritized as candidate biomarkers for downstream protein validation.

To account for the intra-patient correlation inherent in partitioning spatial spots, the four candidate genes were re-evaluated using a linear mixed-effects framework. Spot-level gene expression was modelled using the fixed effect of the clinical recurrence status, with patient identity included as random intercepts. This approach controls for the possible inflation of effective sample sizes by treating individual patients as the primary unit of biological replication.

#### 2.3.7 Validating transcriptionally-defined candidate recurrence genes through immunohistochemical staining of proteins

For IHC staining of candidate proteins, FFPE cancer tissue slides (for FMO2) or tissue microarray slides (for MARCO, PNPLA3 and PAEP) were deparaffinized in xylene and rehydrated in ethanol series, and heat induced antigen retrieval was performed with either Sodium Citrate Buffer (10mM Sodium Citrate, 0.05% Tween 20, pH 6.0, at 100C for 10 minutes, for FMO2), or Diva pH6 decloaker buffer (Cat# DV2004, Biocare medical, CA, USA, at 100C for 20 minutes, for PNPLA3, PAEP and MACO). Sections were then incubated with the following antibodies: Anti-MARCO polyclonal antibody (Cat#PA5-64134, Invitrogen, 1:1000, 90 mins at RT), anti-PNPLA3 polyclonal antibody (Cat#PA5-18901, Invitrogen, 1:500, 90 mins at RT), anti-PAEP polyclonal antibody (Cat#PA5-54152, Invitrogen, 1:500, 90 mins at RT) and anti-FMO2 polyclonal antibody (Cat#ab95977, Abcam, 1:500, 120 mins at RT). Then, the samples were rinsed twice with PBS for 5 minutes, and endogenous peroxidase activity was blocked in 2.5% hydrogen peroxide for 10 minutes at room temperature. The samples were rinsed again in PBS, and incubated for 1 hour at RT with secondary antibodies: MACH – 1 universal polymer (Biocare medical, 30 minutes at RT, for MARCO and PAEP), Goat Probe and polymer (Biocare Medical, 20 minutes at RT for PNPLA3) and biotinylated anti rabbit secondary antibody (Thermofisher, 2 hours at RT, for FMO2). Streptavidin-hourseradish peroxidase was added to samples stained for FMO2 (20 minutes at RT). Diaminobenzidine (Abcam) was used as substrate, and sections were counterstained with haematoxylin. Slides were imaged with a microscope (Olympus BX60) and processed by ImageJ. Cellular scoring was conducted on a semi-quantitative four-point scale by a qualified anatomical pathologist, assessing IHC intensity as follows: negative (0), mild positivity (1), moderate positivity (2), and strong positivity (3).

#### 2.3.8 Validating candidate recurrence genes with external TCGA bulk RNA-seq data

Bulk RNA-seq data were obtained from TCGA-HNSC using the following criteria: (1) diagnosis of primary HNSC without prior treatment, (2) availability of RNA-seq data from the primary tumor sample, and (3) complete data for age, gender, race, alcohol use, and smoking exposure. Using additional patient follow-up information from the TCGA database, patients were classified into two groups: the recurrence group (patients who experienced tumor progression or recurrence during follow-up) and the non-recurrence group (patients with no evidence of progression or recurrence and who were tumor-free at last contact). Age was further categorized into four groups: 0–39, 40–59, 60–79, and ≥80 years. Details on the dataset are provided in **Supplementary Table 2**.

Because the TCGA data represents deeply sequenced, standard bulk RNA counts, gene expression analysis was performed using DESeq2 (38), comparing recurrence and non-recurrence groups while adjusting for all relevant confounding factors mentioned above.

#### 2.3.9 Validating candidate recurrence genes with independent spatial transcriptomics datasets

Marker gene expression was analyzed using two additional public Visium datasets from HNC. The first dataset comprised paired samples collected before and after cancer, and the second consisted of a sample from a patient with no evidence of recurrence after a two-year MRI follow-up [relapse, details at (39)]. In addition, Xenium data from an HNC patient with unknown recurrence status were analyzed to validate the cell-type specificity of *PAEP*. Gene expression values were normalized using the SCT method (27). Cell type identities for the Xenium dataset were determined via automated label transfer using a reference single-cell RNA-sequencing (scRNA-seq) dataset from Quah et al. (40). Following log-normalization of the Xenium transcript counts using the *NormalizeData* function, we implemented the Seurat integration workflow. Specifically, ‘anchors’ were identified between the reference and query datasets using CCA, and cell type labels were projected onto the Xenium data using the *TransferData* function. This approach assigns a prediction score to each cell, ensuring that spatial cell type assignments are consistent with established transcriptomic profiles.

#### 2.3.10 Public cohort survival analysis

To evaluate the prognostic relevance of PAEP expression in a larger clinical context, a formal survival analysis was performed using the KM-plotter web portal (Győrffy, 2024), which integrates public transcriptomic and clinical datasets for head and neck squamous cell carcinoma. Patient cohorts were stratified into high- and low-expression groups based on PAEP mRNA levels using the auto-select best cutoff feature. Overall survival (OS) was evaluated at two distinct clinical endpoints: a short-term, high-risk window restricted to 30 months follow-up, and an unconstrained long-term follow-up extending up to 200 months. Hazard ratios (HR) with 95% confidence intervals (CI) and log-rank p-values were calculated automatically by the portal interface.

## 3 Results

### 3.1 Cell type and cell-type specific gene signature changes between recurrent and non-recurrent patients

**Figure 2:**
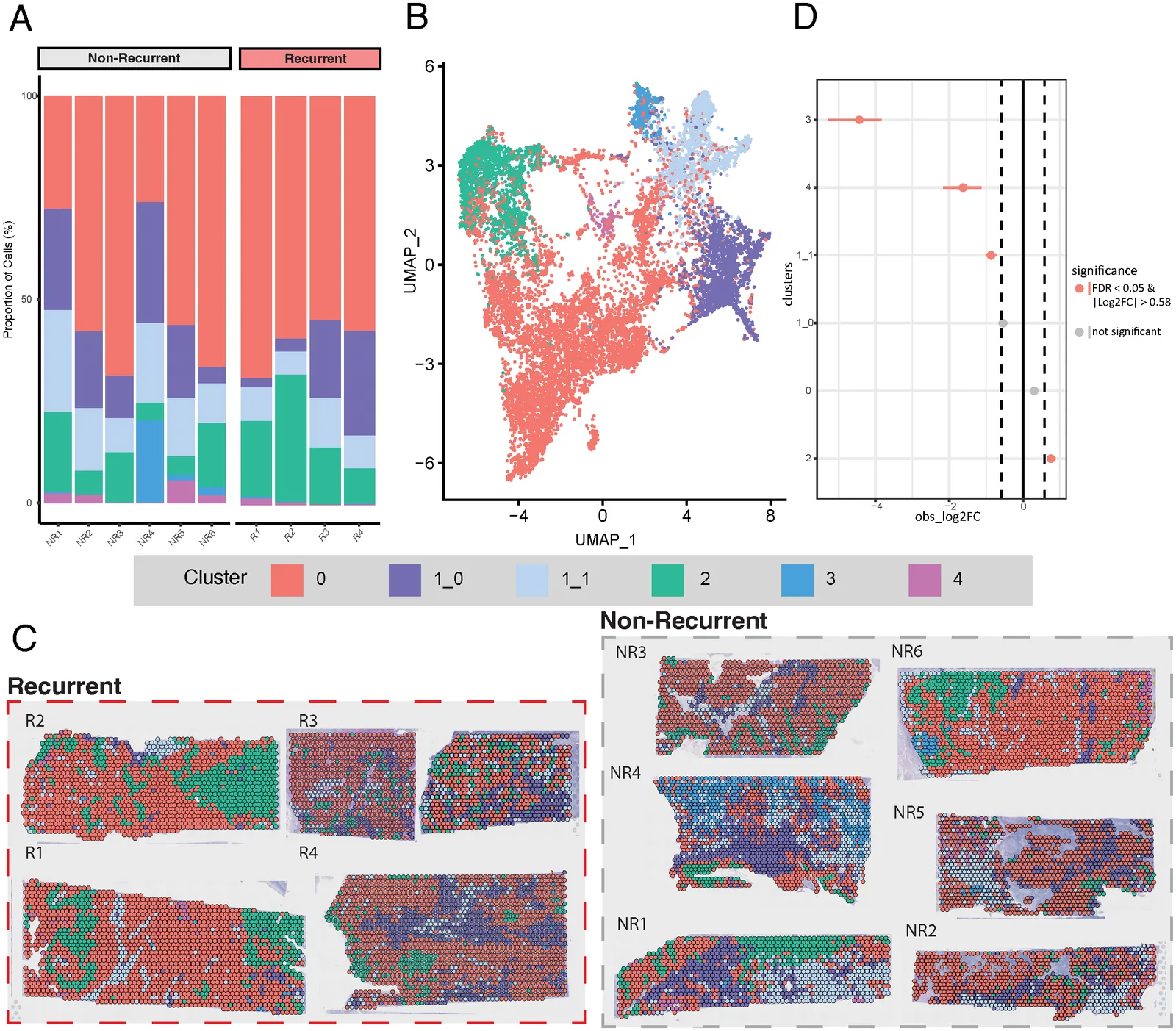
Unsupervised clustering of Visium spots identifies cell groups with potential differential expression between recurrent and non-recurrent HNC samples. **(A)** Stacked bar graph of the proportions of clusters mapped onto each sample. **(B)** UMAP plot of all spots colored based on unsupervised clustering analysis. **(C)** Spatial representation of the clusters mapped onto H&E images for each sample. Colors in **(A**, **B, D)** represent the clusters identified. Cluster 1 was sub-clustered and is displayed here as clusters 1_0 and 1_1. FC = fold change. **(D)** Relative difference in distribution of clusters in recurrent samples compared to non-recurrent samples. The clusters that are significantly different (FDR < 0.05, and |log_2_FC|>0.58) are highlighted in red (permutation test; n=1, 000).

To ensure the reliability of the spatial analysis, we first performed data preprocessing and normalization. Given the high degree of tissue complexity and structural diversity in HNC, as documented by expert pathologist annotations (**Supplementary Figures 1A–C**), robust batch correction was essential to mitigate technical bias and ensure that the resulting signals reflected true biological variation across samples (**Supplementary Figures 2A–D**). Following these quality control measures, we combined unsupervised analyses with histological annotation to organize the intricate histological landscape into biologically meaningful molecular units. We performed unsupervised clustering of all Visium spots based on gene expression similarities, which identified five distinct clusters (**Figures 2A, B**).

Spatial mapping revealed that Clusters 0 and 2 were predominantly localized to malignant areas, showing high spatial concordance with pathologist-defined tumor regions (**Figure 2C**; **Supplementary Figure 1**). This alignment was quantitatively confirmed, with 70.7% of spots in Cluster 0 and 76.7% in Cluster 2 falling within annotated tumor boundaries (**Supplementary Figure 3**). In contrast, Cluster 1 exhibited a heterogeneous distribution, spanning both stromal and immune-enriched regions. This mixed identity was reflected in its enriched GO terms, which broadly combined stromal terms (e.g., *collagen fibril organization*) with general immune functions (e.g., *complement activation*) (**Supplementary Figure 4A**).

To resolve this heterogeneity, Cluster 1 was subclustered into two distinct spatial domains: Subcluster 1_0 and Subcluster 1_1 (**Supplementary Figure 4B**). This separation successfully deconvoluted the biological signals: Subcluster 1_0 localized to immune-rich areas and displayed distinct innate and adaptive immune signatures (e.g., *regulation of B cell activation*), whereas Subcluster 1_1 aligned with the stroma and was enriched for connective tissue functions (**Supplementary Figures 3**, **4C**). Notably, the specific adaptive immune pathways detected in Subcluster 1_0 were obscured when analyzing Cluster 1 as a whole, highlighting the necessity of subclustering to capture the distinct immune microenvironment.

To identify spatial domains associated with clinical outcome, we applied permutation testing and bootstrap analysis (**Figure 2D**). We found that Subcluster 1_1, Cluster 3, and Cluster 4 were significantly enriched in non-recurrent samples, whereas Cluster 2 was enriched in recurrent samples. Subcluster 1_1 (log_2_FC < -0.58), identified above as a stromal compartment, showed significant enrichment in the non-recurrent group, consistent with a role for stromal architecture in tumor containment. Cluster 3 showed the strongest nominal enrichment (log_2_FC = –4.44) and was defined by muscle-related terms such as ‘*striated muscle contraction*’ (**Supplementary Figures 4D, E**). Pathologist annotations localized this cluster predominantly to samples NR4 and NR6, both of which are mandible tumors, suggesting this signature reflects adjacent muscle tissue captured in these specific resections rather than a tumor-intrinsic mechanism. Cluster 4 (log_2_FC = –1.62) also favored non-recurrent samples. Although this cluster contained relatively few spots, it mapped predominantly to immune-rich regions and was associated with the negative regulation of leukocyte migration. Conversely, Cluster 2, which showed enrichment in recurrent samples (log_2_FC > 0.58), mapped largely to tumor and normal mucosal regions. This cluster was characterized by nine enriched GO terms, including immune-related processes (e.g., acute inflammatory response, regulation of humoral response) as well as pathways linked to cell proliferation (**Supplementary Figures 4D, E**).

**Figure 3:**
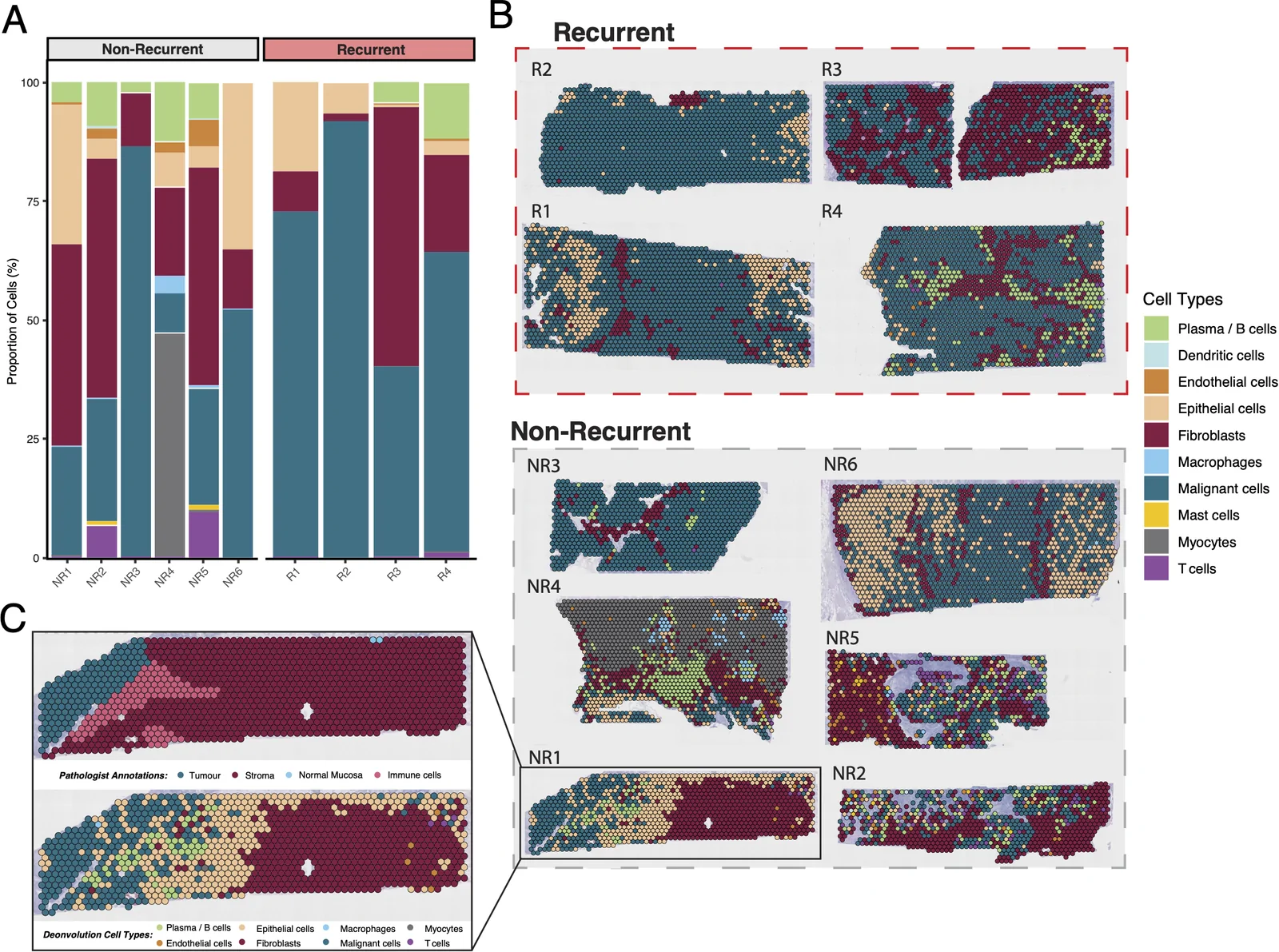
Visium spot deconvolution uncovers varying proportions of HNC-specific cell types between recurrent and non-recurrent samples. **(A)** Stacked bar graph of the proportions of different cell types present within each sample identified through CARD deconvolution. **(B)** Spatial representation of the deconvolution results displaying the most prominent cell type found within each spot. **(C)** Compares the deconvolution results of sample P9900 to the pathologist annotations. Colors indicate histopathological regions defined by pathological annotation (upper) and the corresponding dominant cell types estimated by deconvolution (lower), demonstrating spatial concordance between the two methods.

To further resolve the cellular composition within individual Visium spots and characterize the heterogeneity of the tumor microenvironment, we performed transcriptomic deconvolution, which suggested a divergent immune landscape between outcomes. Non-recurrent samples displayed a trend toward higher immune infiltration, particularly regarding T and B cell populations (**Figure 3A**). Quantitatively, both the average proportion of the two lymphocytes per spot (**Supplementary Figure 5A**) and the frequency of immune-dominated spots (**Supplementary Figure 5B**) were elevated in the non-recurrent group relative to recurrent samples. However, these differences did not reach statistical significance (Wilcoxon test, p > 0.05), likely reflecting the limited statistical power of the cohort size. Despite this, the spatial distribution of these estimated cell types aligned closely with pathologist-annotated regions, reinforcing the biological validity of the deconvolution signal (**Figures 3B, C**; **Supplementary Figure 6A**). For instance, the ‘myocyte’ signal observed within histologically benign salivary tissue accurately captures endogenous myoepithelial cells, which physiologically surround salivary acini and share strong transcriptional overlap with muscle lineages, a biological nuance consistent with the cluster definitions in our reference atlas. For sanity check, we confirmed that the tumor compartment demonstrates clear and robust enrichment for epithelial markers, including *KRT5*, *KRT14*, *TP63*, and *EPCAM*. Conversely, the stroma regions display expected enrichment for the canonical mesenchymal/stromal marker *VIM* and *VWF* (**Supplementary Figure 6B**). However, because these spatial resolution maps are computationally inferred by CARD rather than directly observed, they represent statistical cell-type proportions within each multicellular spot.

Spatial analysis highlighted a key distinction in immune topography: within non-recurrent tumors, T cells and B cells were frequently observed colocalizing in the same histological niches. In contrast, recurrent samples tend to lack both lineages (**Supplementary Figures 7A–D**), suggestive of an immune-excluded phenotype. This trend was further confirmed by quantitative density profiling: while recurrent tumors displayed a single density peak near zero for both cell types, non-recurrent samples revealed a distinct secondary peak, representing a specific subpopulation of tumor spots containing approximately 5% T cells and 1% B/plasma cells (**Supplementary Figure 8**). These results indicate that the coordinated infiltration of both T and B cells into the tumor core is a specific feature of non-recurrent disease, distinguishing it from the immunologically quiescent environment of recurrent tumors.

### 3.2 Spatial DE analysis of cell types identified genes linked to recurrence in HNC

**Figure 4:**
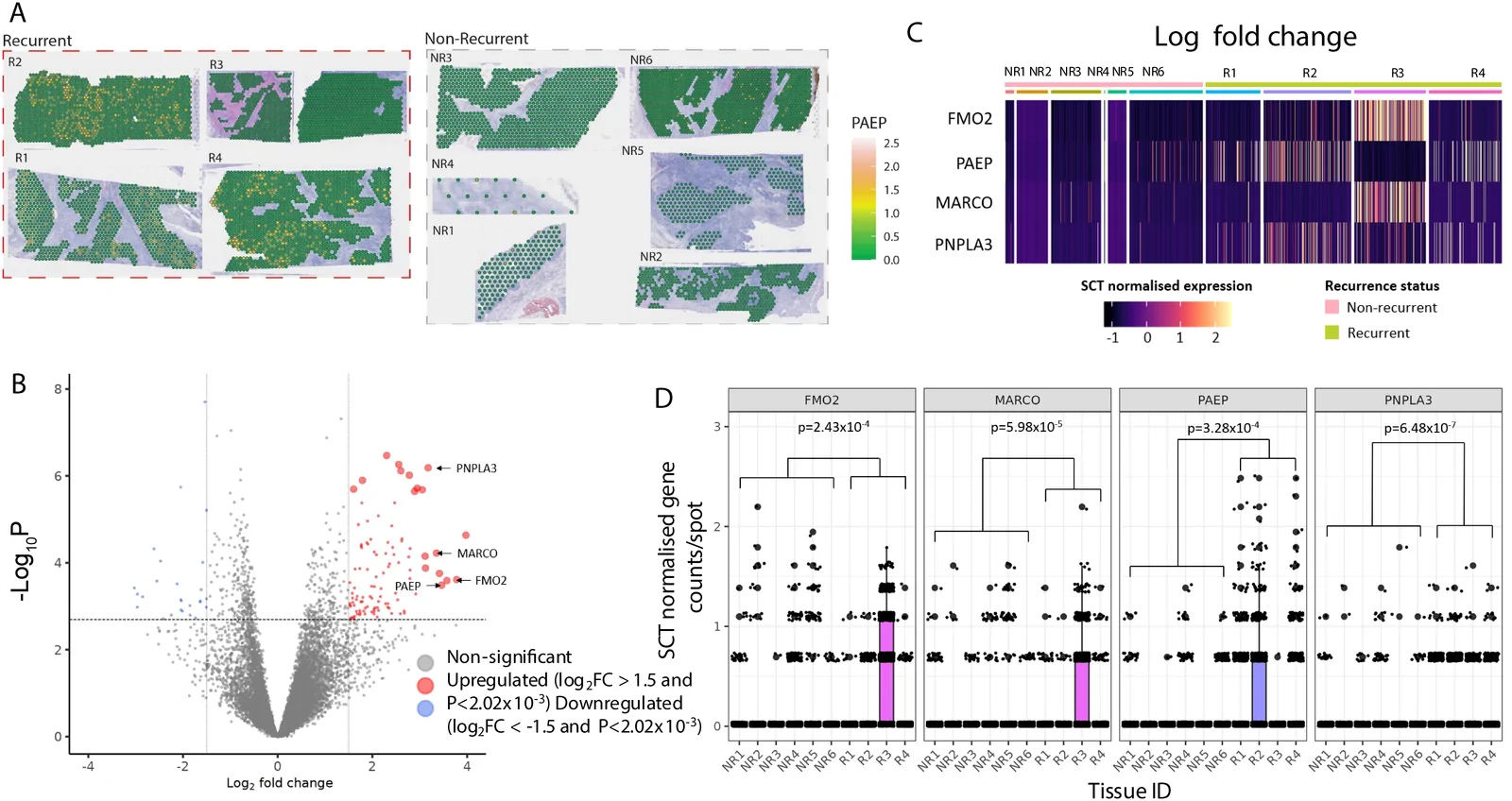
Analysis of differential gene expression in recurrent HNC tumor regions to identify potential genes for recurrence. **(A)** Gene expression of biomarkers of recurrence, *PAEP* mapped on tumor spots H&E image. Tissue samples were grouped by recurrence (left) and non-recurrence (right). Only the spots annotated as tumor are shown, and all gene counts have been SCT normalized. **(B)** Volcano plot of tumor recurrence DE analysis results with upregulated (log_2_FC > 1.5 and P<2.02 ×10^-3^) and downregulated (log_2_FC < -1.5 and P<2.02 ×10^-3^) genes highlighted in red and blue dots respectively. The 18 upregulated DEGs that had the highest log2FC and/or p-value, and these were considered for protein validation, and are presented as large red data points. FMO2, *PAEP*, MARCO and PNPLA3 are annotated as the four candidate genes that were subsequently stained for protein validation. All p-values were Benjamini-Hochberg (BH)-adjusted. **(C)** Heatmap of FMO2, *PAEP*, MARCO and PNPLA3 gene expression across 10 patient samples. **(D)** Boxplot of SCTransform normalized gene counts per spot across all non-recurrent (NR) and recurrent (R) tissues. The gene expression of each spot is superimposed on the box plots to highlight the density of gene expression across tissues. The p-value between NR and R samples in total for each gene is shown.

To identify candidate biomarkers associated with HNC recurrence, we focused our comparative analysis specifically on the malignant compartment (**Figure 4A**). We isolated spatial gene expression data for spots located within pathologist-annotated tumor regions (**Supplementary Figure 2A**) and performed DE analysis to contrast the transcriptional profiles of recurrent versus non-recurrent tumors. We compared 13, 266 genes from 26 pseudo-samples (see Methods; also refer to the linear mixed-effects approach) and identified 271 and 265 genes significantly up- and downregulated in recurrent tumor spots compared to non-recurrent tumor spots (P<2.02×10^-3^), respectively (**Figure 4B**).

To explore functional pathways associated with tumor recurrence, we first performed GSEA using the Reactome database (41). Non-recurrence samples showed upregulation of MAPK6/MAPK4 and PCP/CE pathways (**Supplementary Figure 9**), consistent with the context-dependent pro- and anti-oncogenic roles of these kinases (22) and the PCP/CE pathway (42). Broad GO enrichment analysis of tumor DEGs did not identify significant terms, likely due to disease heterogeneity.

To address this, recurrence DEGs were stratified by p16 status. In p16-positive tumor regions, pseudo-bulk analysis identified 1, 486 upregulated and 1, 677 downregulated DEGs (filtering threshold P < 1.12×10^-2^), whereas p16-negative regions had 231 upregulated and 404 downregulated DEGs in recurrent samples (P < 2.14×10^-3^) (**Supplementary Figures 10A, B**). Stratification revealed significant GO term enrichment (P < 0.05), including: p16-positive upregulated DEGs were enriched for collagen fibril organization and xenobiotic metabolism, while downregulated DEGs were associated with metabolic changes. In p16-negative recurrent tumors, downregulated DEGs were enriched for viral regulation, IFN-β responses, and apoptosis, while no upregulated DEGs were found (**Supplementary Figure 10C**). Clustering of enriched GO terms suggested that p16-positive and p16-negative tumors may use distinct pathways to drive recurrence (**Supplementary Figure 10D**).

Analyses of stromal regions (**Supplementary Figure 11**) and stromal–TIL–tumor interfaces (**Supplementary Figure 12**) identified additional genes significantly upregulated in recurrent tumors. These recurrence-associated markers showed enrichment in proliferation, protein processing, and immune regulatory pathways. Although limited by the small cohort size, these findings in the stroma and interface compartments are broadly consistent with the immune-suppressed and proliferative profile observed within the recurrent tumor regions.

### 3.3 Identification and validation of candidate genes associated with tumor recurrence

To find top recurrence candidate biomarkers, we filtered over 200 upregulated DEGs (**Supplementary Table 3**) in recurrent tumor samples, narrowing it down to 104 DEGs with a fold change (FC) threshold (|log_2_FC|>1.5) (**Figure 4B**). The top 10 genes, ranked by fold-change and p-value, underwent systematic spatial evaluation to verify their distribution. Candidates were defined as ‘robust’ if they demonstrated consistent upregulation within the tumor compartments of at least 50% of the recurrent samples, ensuring that the prioritized markers represented the broader recurrent phenotype rather than isolated outliers.

For instance, *CWH43* was excluded despite a high fold-change (log_2_FC = 3.97) because its expression was restricted almost exclusively to sample R2. In contrast, *FMO2, PNPLA3, PAEP*, and *MARCO* emerged from this curation as the most robust candidates for further validation, though they exhibited distinct patterns of heterogeneity (**Figures 4C, D**). Specifically, *PAEP* and *PNPLA3* demonstrated strong, consistent upregulation across samples R1, R2, and R4. Conversely, the signals for *FMO2* and *MARCO* were predominantly driven by sample R3, reflecting a distinct transcriptional profile in this tumor. Importantly, while the expression of *FMO2* and *MARCO* appeared lower in R1, R2, and R4 compared to R3, it remained consistently elevated relative to the non-recurrent baseline. By retaining both sets of markers, we aimed to account for inter-tumor heterogeneity and ensure detection across diverse recurrence phenotypes.

**Figure 5:**
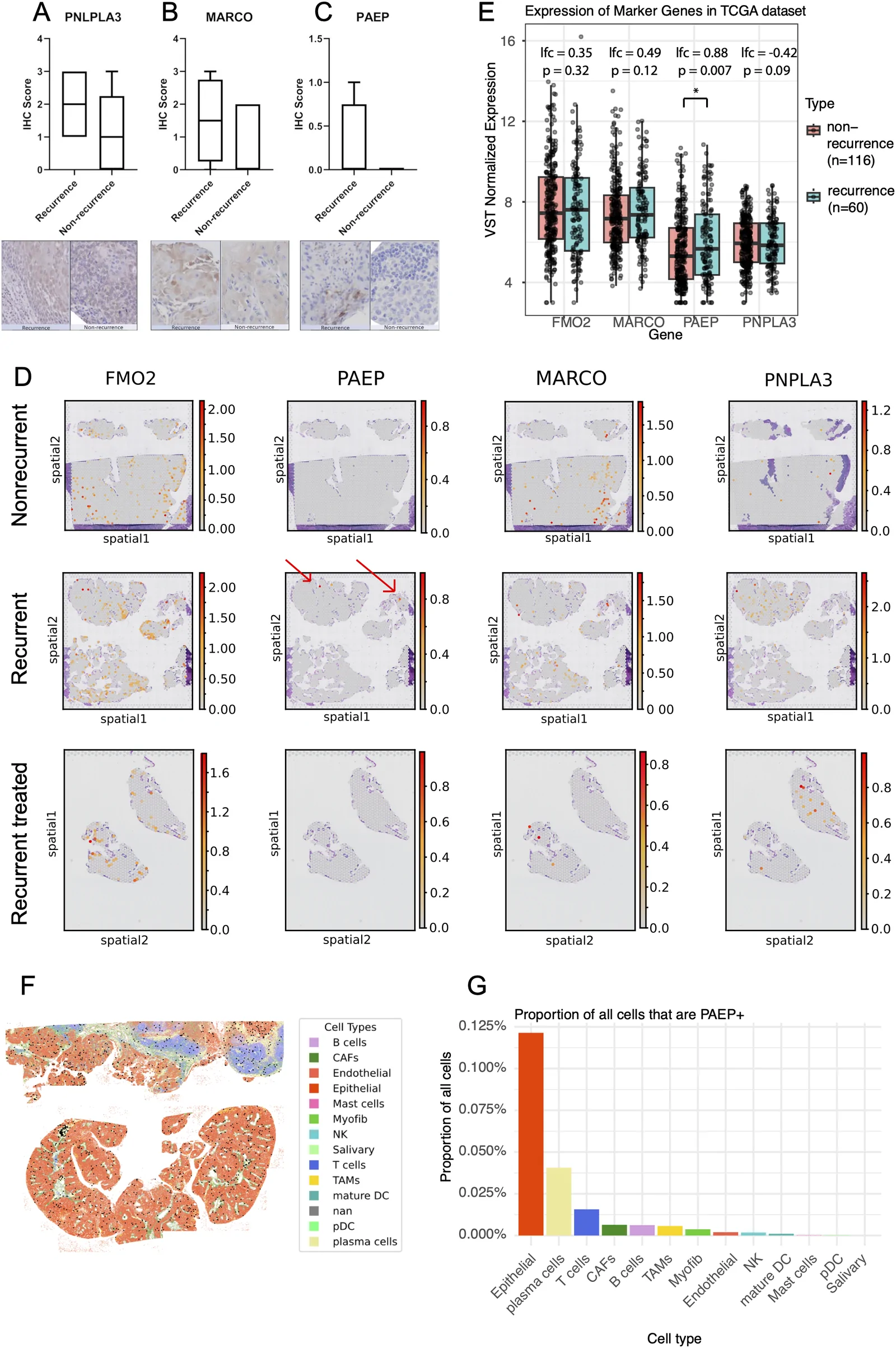
Validation of candidate recurrence-associated genes. **(A–C)** Protein-level validation: box-and-whisker plots of IHC scores and representative immunohistochemistry images for **(A)** PNPLA3, **(B)** MARCO, and **(C)** *PAEP* in tumor regions. **(D)**Validation in an external spatial transcriptomics dataset. The panel compares gene expression across three distinct clinical groups to assess association. Rows labeled ‘Recurrent’ represent baseline primary tumors from patients who later developed recurrence (pre-relapse). ‘Recurrent treated’ represents the actual recurrent tumor harvested after treatment failure from the same patients (post-relapse). ‘Non-recurrent’ represents primary tumors from patients who remained disease-free. Orange arrows indicate spots with significant PAEP expression, which are uniquely restricted to the baseline tumors of the recurrence-prone group. **(E)** Gene expression comparison in TCGA-HNSC samples between recurrence (n = 60) and non-recurrence (n = 116) groups. The y-axis shows variance-stabilized transformed expression. Differential expression statistics are displayed above each boxplot, with “lfc” indicating log2 fold change and “p” the p-value. **(F)** Spatial visualization of *PAEP* expression and cell-type locations in HNC Xenium data. Black dots represent detected *PAEP* transcripts and all epithelial cells here are malignant. **(G)** Proportion of cell types among *PAEP*-expressing cells in Xenium data.

Protein-level validation was performed by IHC on tumor regions from 4 patients with recurrence and 6 without recurrence. Representative images are shown in **Figures 5A–C**. *PNPLA3* expression was higher in the recurrence group (scores 1–3, median 2) compared to the non-recurrence group (scores 0–3, median 1). *MARCO* expression was also elevated in patients with recurrence (scores 0–3, median 1.5) relative to those without recurrence (scores 0–2, median 0). *PAEP* expression was low overall, with scores of 0–1 (median 0) in the recurrence group, and uniformly negative in the non-recurrence group.

To further assess the association between candidate gene expression and tumor recurrence, we examined published spatial transcriptomics data comparing recurrent and non-recurrent HNC samples. Among the four markers, *PAEP* exhibited the most distinct association with recurrence status (**Figure 5D**; **Supplementary Table 4**). Notably, distinct *PAEP*-positive spots (highlighted in orange) were exclusively detected in the baseline primary tumors of patients who subsequently relapsed (‘Recurrent’ group). In contrast, PAEP expression was absent in both the matched post-treatment recurrences (‘Recurrent treated’) and the primary tumors of patients who remained disease-free (‘Non-recurrent’). This specific localization to the pre-treatment state of high-risk patients suggests that PAEP may serve as a candidate biomarker associated with recurrence risk. Similarly, *PNPLA3* showed elevated expression specifically in the pre-relapse primary tumors compared to the other clinical groups.

**Figure 6:**
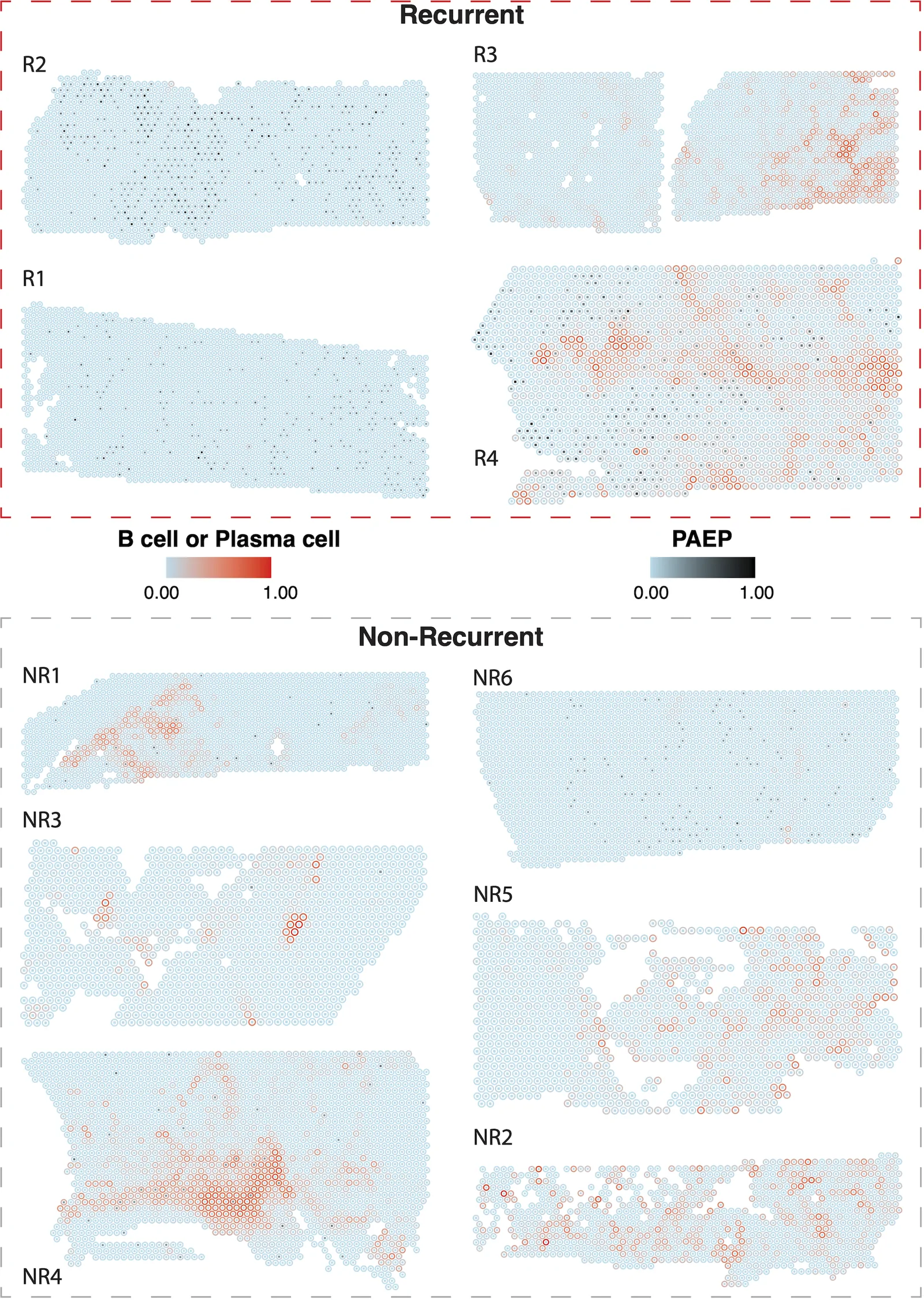
Visualization of *PAEP* expression and B-cell/plasma cell proportions in Visium spots for each sample. For each spot, the edge color of the circle represents the relative abundance of B cells and plasma cells, while the center square color represents relative *PAEP* expression. Both values are scaled to the maximum across all samples (1 = highest expression or cell proportion, 0 = lowest). NR, Non-recurrent; R, Recurrent.

For generalization, we also analyzed bulk RNA-seq data from 60 recurrence tumor samples and 116 non-recurrence tumor samples obtained from the TCGA database. After statistically adjusting for potential confounding factors, including primary tumor anatomical site, age, gender, race, and history of exposure to tobacco and alcohol, the expression of *PAEP* remained significantly elevated in the recurrence group compared to the non-recurrence group log_2_FC = 0.88, p = 0.007; **Figure 5E**), supporting its potential role as a biomarker for recurrence. Because this analysis was designed specifically to test the directional hypothesis of our four pre-selected candidate genes rather than to perform an exploratory genome-wide screen, unadjusted p-values were utilized. This association was not observed for the other three genes examined, which may be due to the lack of cell type specificity inherent in bulk RNA-seq data (i.e., expression signals from tumor cells may be diluted by non-tumor cells present in the sample). In contrast, *PAEP* expression in HNC is largely confined to cancer cells, as indicated by an external Xenium data which shows *PAEP* expression at single-cell resolution (**Figures 5F, G**). This aligns with Human Protein Atlas reports showing minimal *PAEP* expression in non-glandular and non-luminal normal cell types, underscoring the value of single-cell and spatial transcriptomics for identifying cell type–specific markers. Interestingly, plasma cells and T cells emerged as the next most prominent *PAEP*-expressing populations (**Figure 5G**), some of which can have higher *PAEP* expression than cancer cells (**Supplementary Figure 13**), suggesting a potential functional role for *PAEP* within immune compartments. Leveraging spatial transcriptomics, we observed a strong negative colocalization of *PAEP* with B cells and/or plasma cells in nine of ten HNC samples (**Figure 6**), whereas little spatial association was detected with T cells (**Supplementary Figure 14**).

To assess the relevance of these spatial findings to clinical outcomes, we analyzed *PAEP* expression within an integrated public HNC cohort via the KM-plotter analytical database. Given that a 30-month threshold effectively captures the majority of high-risk HNC recurrence events (Haring, 2023), we evaluated survival within this clinically relevant window. High *PAEP* expression was significantly associated with reduced survival within this timeframe (HR = 1.57 [1.14 to 2.15], logrank P = 0.0049; **Supplementary Figure 15A**), whereas the prognostic signal was attenuated over long-term follow-up (HR = 1.18, logrank P = 0.27; **Supplementary Figure 15B**). This association supports *PAEP* as a potential marker of early treatment failure, providing clinical context that aligns with our recurrence-focused spatial discovery cohort. Because comprehensive recurrence-free survival data were unavailable in this public dataset, overall survival was utilised as a proxy metric. This endpoint includes all-cause mortality, which may introduce confounding variables unrelated to disease recurrence. Additionally, the automated cut-off selection used to stratify expression groups introduces statistical optimism and may overestimate the significance of the survival difference. These retrospective results represent a baseline characterization of a candidate feature rather than a validated clinical prognostic tool.

To evaluate the statistical robustness of the four selected candidate genes against intra-patient correlation, we re-analysed the spatial spot data using a linear mixed-effects model with patient identity specified as a random effect. Under this framework, *PAEP* emerged as the only candidate to retain statistical significance (p-value threshold 0.05), demonstrating a positive association with the recurrent phenotype (effect size = 0.107, p = 0.048, **Supplementary Table 5**). The statistical signals for *MARCO*, *PNPLA3*, and *FMO2* did not meet the threshold for significance after accounting for patient-level variance. This computational outcome aligns with our downstream validation experiments.

## 4 Discussion

This study produced a valuable data resource of spatially resolved transcriptomics to contrast baseline tumor characteristics between multiple recurrent and non-recurrent HNC samples. In contrast to conventional bulk RNA-seq, spatial transcriptomics enables the resolution of gene expression patterns within defined tumor compartments, thereby enhancing biomarker discovery specificity by reducing noise from surrounding non-tumor signals. By separately interrogating tumor and stromal regions annotated by the pathologist, we provide high-resolution insights into the cellular and molecular determinants of recurrence risk.

Spatial analysis suggested a trend toward a higher abundance of immune cells in non-recurrent tumors, with a clear enrichment of B cells and plasma cells. While the prognostic value of T-cell infiltration, especially CD8^+^ T cells, is well established across solid tumors, including HNC (43–47), the contribution of B cells and plasma cells has been less clear. Our exploratory findings align with emerging evidence that B cells and plasma cells may enhance antitumor immunity (48, 49). Patients without recurrence exhibited a trend toward greater immune infiltration within malignant regions, suggesting that coordinated T- and B-cell activity may underlie a more effective endogenous immune response.

Notably, reduced B-cell and plasma cell infiltration was not uniformly associated with recurrence. For example, sample R3 recurred despite high B-cell abundance, possibly reflecting its p16-positive status, as distinct pathway enrichments were observed in p16-positive tumors. This highlights the complexity of recurrence, which is unlikely to be explained by a single factor. Larger cohorts will be needed to identify additional drivers with statistical power, though this comes with the trade-off of balancing depth with breadth of analysis.

Through combined statistical testing and careful evaluation of robustness, we identified tumor-specific DEGs most strongly associated with recurrence. *FMO2*, *PAEP*, *MARCO*, and *PNPLA3* emerged as top candidates. Of these, three exhibited an observational pattern of elevated protein expression in the recurrent group, with further evidence provided by external datasets and published literature.

Among these, *PAEP* was the most notable and statistically significant in the small discovery cohort. *PAEP* encodes glycodelin, a secreted glycoprotein with established roles in reproduction, including the prevention of premature capacitation (50) and the provision of immunoprotection during implantation and placentation (51). Beyond these functions, glycodelin exerts potent immunomodulatory effects. It modulates T-cell responses by raising the activation threshold through interactions with surface molecules such as CD45 (and possibly CD7), thereby skewing cytokine production toward a Th2 profile and inducing apoptosis in activated lymphocytes via a caspase-dependent mitochondrial pathway (52, 53). Glycodelin also suppresses B-cell proliferation and alters monocyte cytokine secretion, collectively fostering an immunosuppressive environment critical for maternal tolerance during pregnancy (54). Literature further suggests that glycodelin’s N-glycosylation–derived oligosaccharide chains may engage CD22 on B cells to mediate immunosuppressive activity (55), though this interaction has not yet been validated in HNC.

In our analysis, *PAEP* expression in tumor regions showed an inversely correlated trend with B-cell and plasma cell abundance, suggesting an immunomodulatory role that could weaken humoral immune responses and contribute to recurrence. This finding is consistent with prior reports of *PAEP* upregulation in immune-resistant metastatic tumors and correlations with immune infiltration and checkpoint expression across cancer types (56). To our knowledge, this spatial comparison suggests the new evidence implicating *PAEP* in shaping the immune microenvironment of HNC, providing a potential mechanistic link between *PAEP* expression, tumor immune evasion, and recurrence risk.

Other recurrence-associated DEGs also point to immune modulation. *FMO2*, previously linked to poor prognosis in ovarian and gastric cancers (57–59), was enriched in activated CAFs and correlated with tumor-associated macrophage infiltration (58). MARCO, a macrophage scavenger receptor, was overexpressed in recurrent HNC and is known to promote tumor progression and immune evasion, with anti-MARCO antibodies showing preclinical promise (43, 60–63). *PNPLA3* remains poorly characterized in cancer; while certain variants predispose to liver cancers and metabolic liver disease (64, 65), its role in extrahepatic tumors remains unclear (66). For these genes, associations with HNC recurrence could not be consistently validated across datasets, likely reflecting the biological complexity of recurrence and the limitation of sample size.

A limitation of this study is the relatively restricted cohort size, consisting of 10 samples (4 recurrent and 6 non-recurrent) distributed across four slide captures. While this sample size limits the statistical power to robustly validate subtle transcriptomic differences or generalize findings across heterogeneous HNC populations, the study was designed to prioritize spatial resolution over cohort breadth. By generating high-dimensional data within defined histological compartments, we were able to identify robust molecular signals in the tumor microenvironment that might be obscured in larger, bulk RNA-seq cohorts.

External datasets were used to validate the findings. Furthermore, the small cohort size combined with high baseline clinical and anatomical heterogeneity precluded the use of strict patient-level pseudo-bulk aggregation due to insufficient degrees of freedom. Consequently, our discovery-phase differential expression analysis relied on intra-tumour pseudo-samples, which introduces intra-patient correlation and likely understates the initial p-values. Additionally, we performed linear mixed effects analysis, simultaneously taking into account inter-patient and intra-patient variation, to provide a comprehensive view of possible statistical approaches.

To mitigate the constraint by a small sample size, the discovery outputs were treated strictly as hypothesis-generating candidate features, with the statistical burden of proof shifted to our independent, patient-level external validation cohort (n = 176) where clinical confounders were controlled. While multivariate analysis of this large-scale external cohort demonstrates that the upregulation of molecular features like *PAEP* occurs independently of anatomical sub-locale, future site-matched spatial transcriptomic cohorts with larger sample sizes are required to decouple generalised recurrence-associated immune architectures from baseline tissue variations.

The linear mixed-effects framework was applied to take into account patient-level variance as applied to assess the four selected candidates. This re-evaluation confirmed *PAEP* as the top candidate marker maintaining statistical significance under a mixed-effects framework, whereas *MARCO*, *PNPLA3*, and *FMO2* did not retain significance once patient-level variation was controlled. This computational result explains our downstream empirical findings, where *PAEP* was the top candidate feature to receive reproducible support in protein-level assessment and external cohorts.

Our findings demonstrate that spatially resolved transcriptomics can suggest cellular and molecular features associated with HNC recurrence, proposing the roles of both tumor-intrinsic markers and the immune microenvironment. By identifying *PAEP* as a candidate feature alongside descriptive observations regarding the contribution of B cells and plasma cells, this work demonstrates the utility of spatially informed approaches for candidate feature discovery. With continued advances in image-based ST and AI-driven analysis (67), such markers hold promise for the development of diagnostic tools that can guide recurrence risk stratification and personalized treatment in HNC.

## Data Availability

The raw human spatial transcriptomic data presented in this study have been deposited in the University of Queensland eSpace repository under DOI: https://doi.org/10.48610/5842caa. These data are not publicly accessible because they contain potentially identifiable human research data and are subject to ethical, institutional, and participant-consent restrictions. Requests for access should be submitted through the UQ eSpace repository and will be considered following approval by the relevant data custodian and institutional ethics requirements. The processed expression matrices, associated metadata, and other derived datasets supporting the conclusions of this article are publicly available in Zenodo at https://doi.org/10.5281/zenodo.18238293. The complete computational analysis pipeline, including preprocessing and spatial analysis scripts, is publicly available on GitHub at Available at: https://github.com/BiomedicalMachineLearning/RecurrenceHN.
