# Supplementary material for: Spatial transcriptomics and histopathological analyses reveal potential biomarkers and immune features linked to recurrence in head and neck cancer
Source: Front Immunol. 2026 Aug 11;17:1713732. doi: 10.3389/fimmu.2026.1713732 (PMC13503330; doi:10.3389/fimmu.2026.1713732)
Supplement: Supplementary file 1 [file DataSheet1.pdf]

Supplementary Material

1 Supplementary Figures

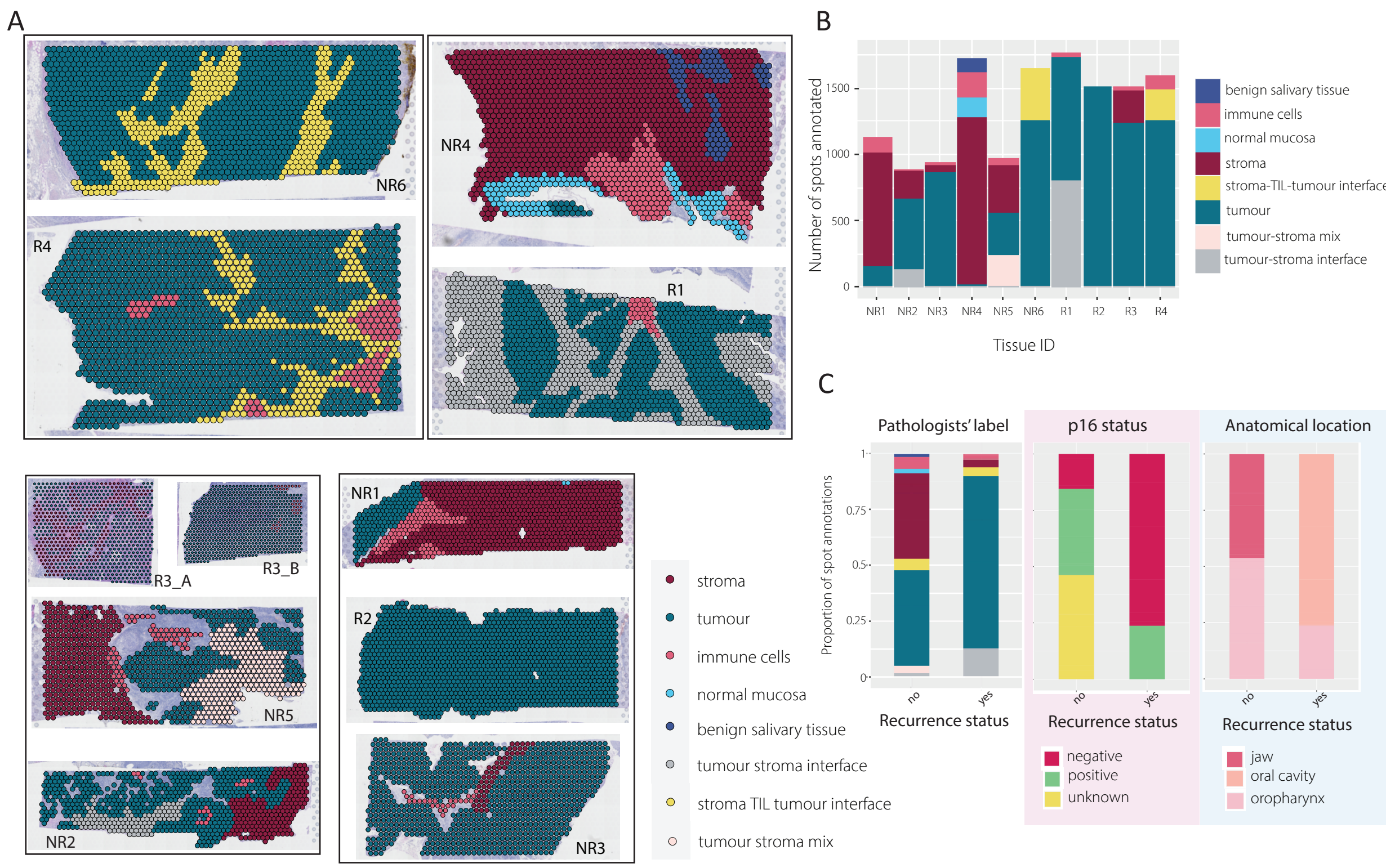

Figure S1. Profile of the tissue samples of the 10 HNSCC patients. (A) Distribution of eight cell types/tissue types identified by pathologist using H&E images. Spots that were not annotated by the pathologist were not considered in the cell type-specific differential gene expression analyses. Tissues are grouped as they were arranged on the capture array, as framed by the black boxes. **(B)** Bar plot of cell types annotated on the spots of each of the 10 tissues. **(C)** Stacked bar plot of spots annotated by cell type (left), p16 status (middle) and tissue location (right) for non-recurrence and recurrence status, respectively.

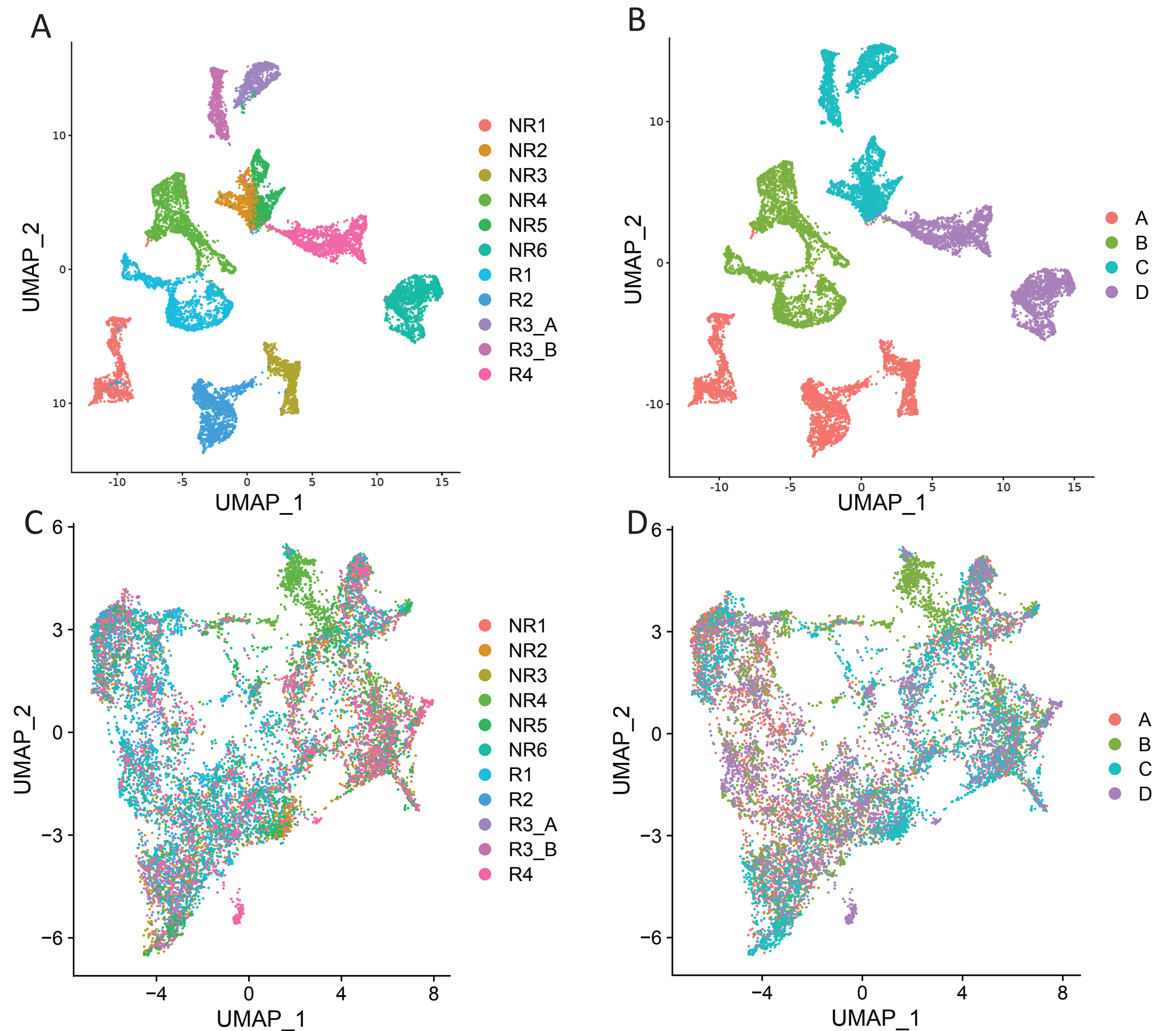

Figure S2. Integration and batch effect removal of Visium samples. UMAP plots colored by tissue sample (left) and capture array (right) **(A, B)** before integration and **(C, D)** after integration. These plots highlight the transition from sample-specific clustering to a unified transcriptomic landscape. Patient R3 provided two tumor blocks (R3_A and R3_B). Sample distribution across arrays: Array A (NR1, NR3, R2), B (NR4, R1), C (NR2, NR5, R3_A, B), and D (NR6, R4).

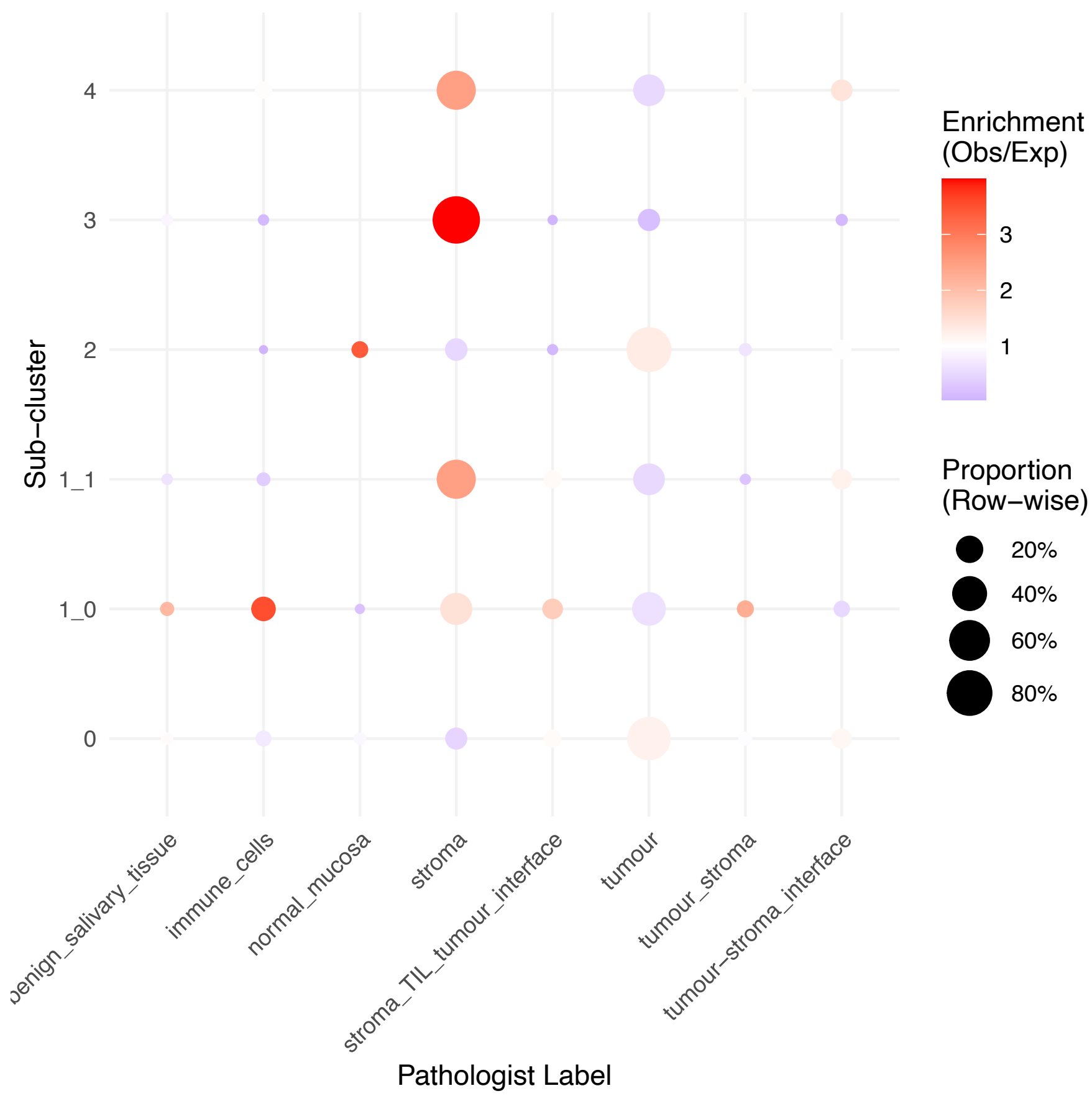

Figure S3. alignment of spot clustering with pathologist annotation. Pathologist labels are shown along the x-axis (columns) versus transcriptome clustering along the y-axis (rows). The area of each bubble corresponds to the mean estimated proportion of a cluster within the specific pathologist-labeled region. The bubble color intensity reflects enrichment of spots within that region comparing to the global background mean proportion.

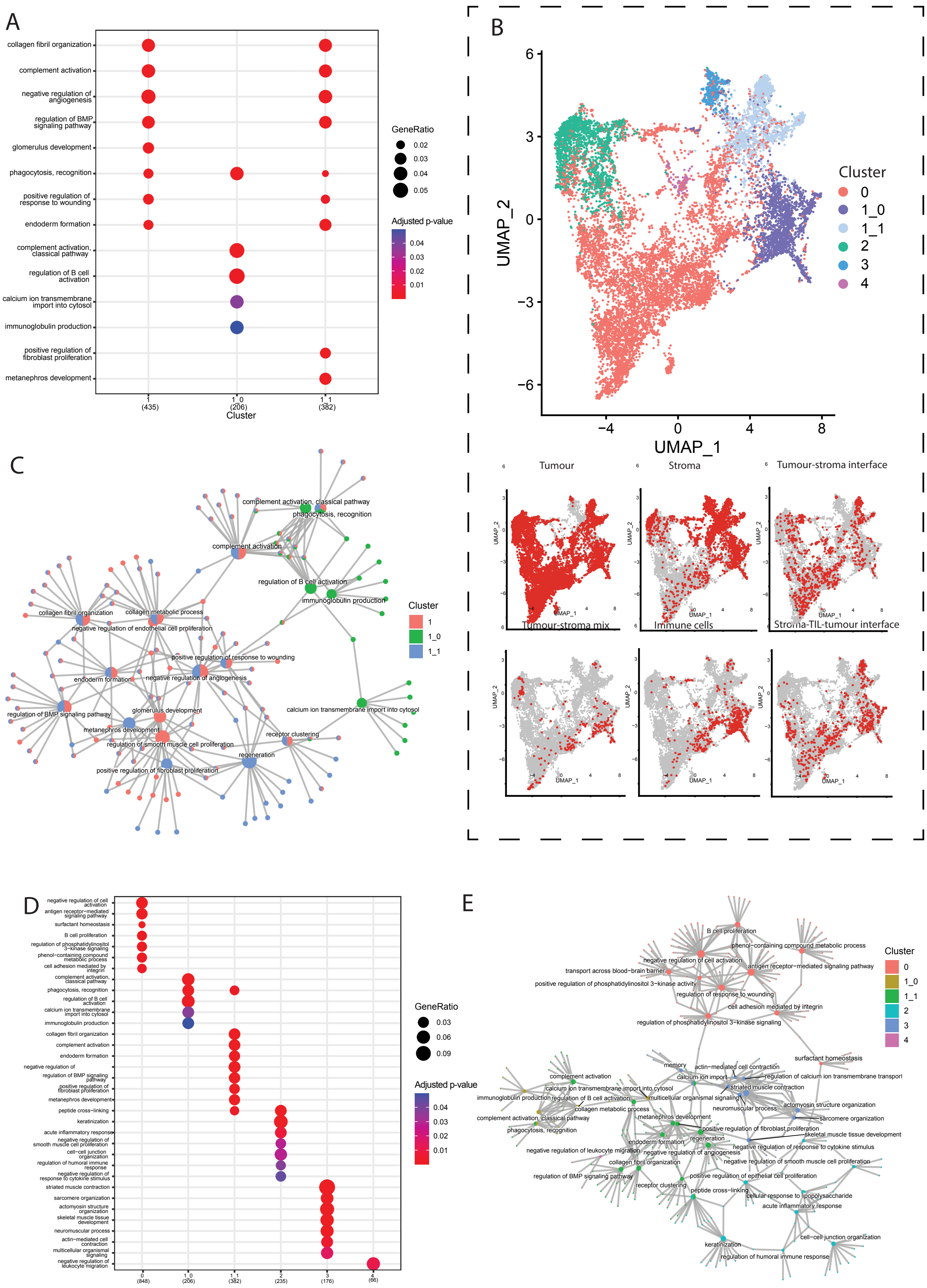
A
collagen fibril organization
complement activation
negative regulation of angiogenesis
regulation of BMP signaling pathway
glomerulus development
phagocytosis, recognition
positive regulation of response to wounding
endoderm formation
complement activation, classical pathway
regulation of B cell activation
calcium ion transmembrane import into cytosol
immunoglobulin production
positive regulation of fibroblast proliferation
metanephros development
1 (435)
1_0 (206)
1_1 (382)
Cluster
GeneRatio
0.02
0.03
0.04
0.05
Adjusted p-value
0.04
0.03
0.02
0.01
B
UMAP_2
UMAP_1
Cluster
0
1_0
1_1
2
3
4
Tumour
Stroma
Tumour-stroma interface
Tumour-stroma mix
Immune cells
Stroma-TIL-tumour interface
C
Cluster
1
1_0
1_1
D
Cluster
0 (848)
1_0 (206)
1_1 (382)
2 (235)
3 (176)
4 (66)
GeneRatio
0.03
0.06
0.09
Adjusted p-value
0.04
0.03
0.02
0.01
E
Cluster
0
1_0
1_1
2
3
4

Figure S4. Unsupervised clustering and functional enrichment analysis. (A) Dot plot showing the top enriched GO terms for main cluster 1 and its constituent subclusters (1_0 and 1_1). **(B)** Comparison of unsupervised clustering (top) and pathologist-annotated cell types (bottom) projected onto integrated UMAP plots. Unsupervised analysis identified five distinct cluster groups, which show high concordance with the six pathologically defined cell types. **(C)** Network plot (enrichment map) of GO terms from panel A; node colors indicate cluster assignment, and edges represent gene overlap between terms. **(D)** Dot plot displaying the top 7 unique enriched GO terms for each main cluster. **(E)** Network representation illustrating the functional relationships between the GO terms shown in (D).

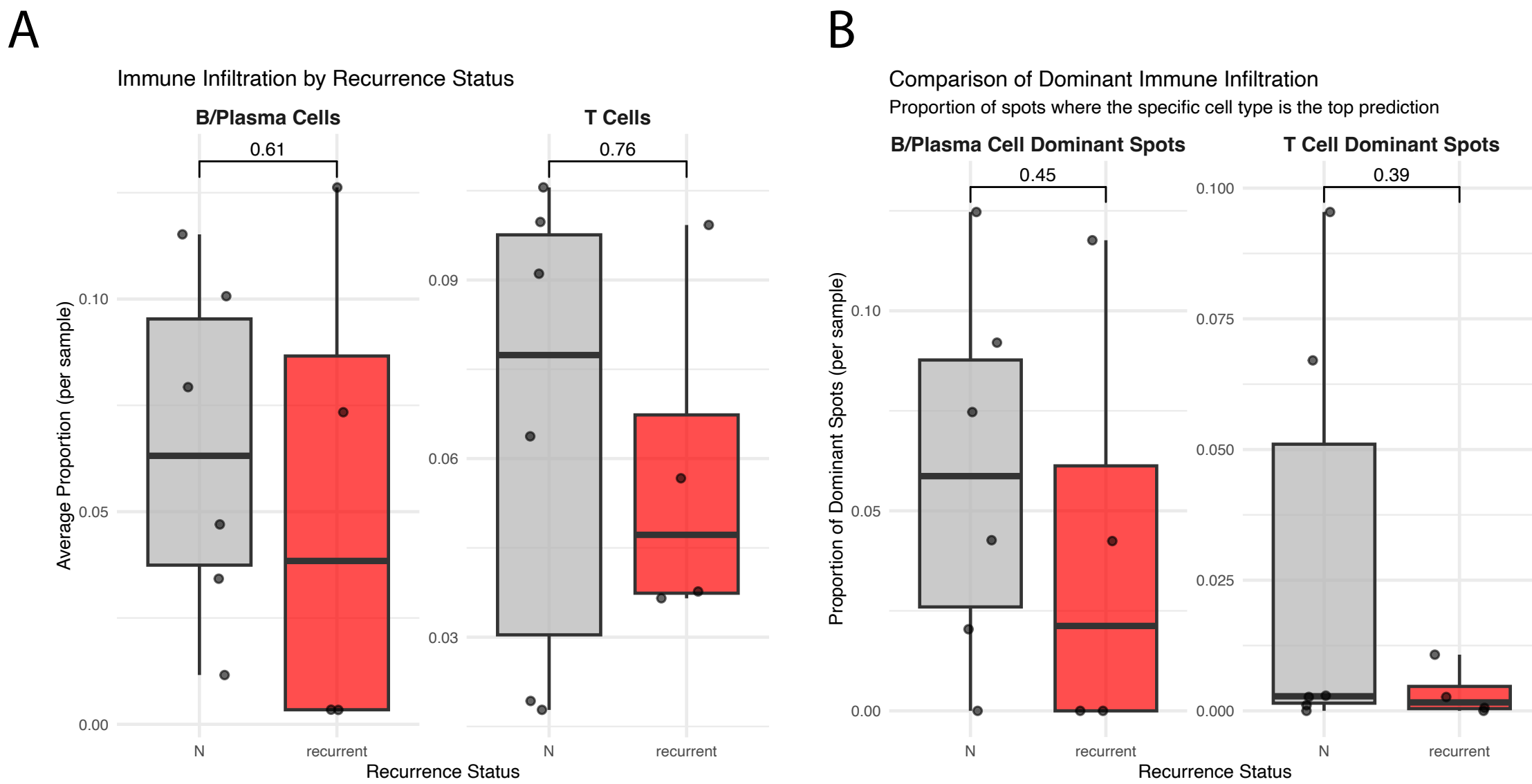

Figure S5. Quantitative comparison of immune infiltration in Visium data. (A) Average proportion of lymphocytes (B/plasma and T cells) per spot across recurrence groups. **(B)** Frequency of spots where B/plasma cells or T cells are the dominant cell type based on transcriptomic deconvolution. Although higher trends are observed in the non-recurrent (N) group, no comparisons reached statistical significance (Wilcoxon test, $p > 0.05$).

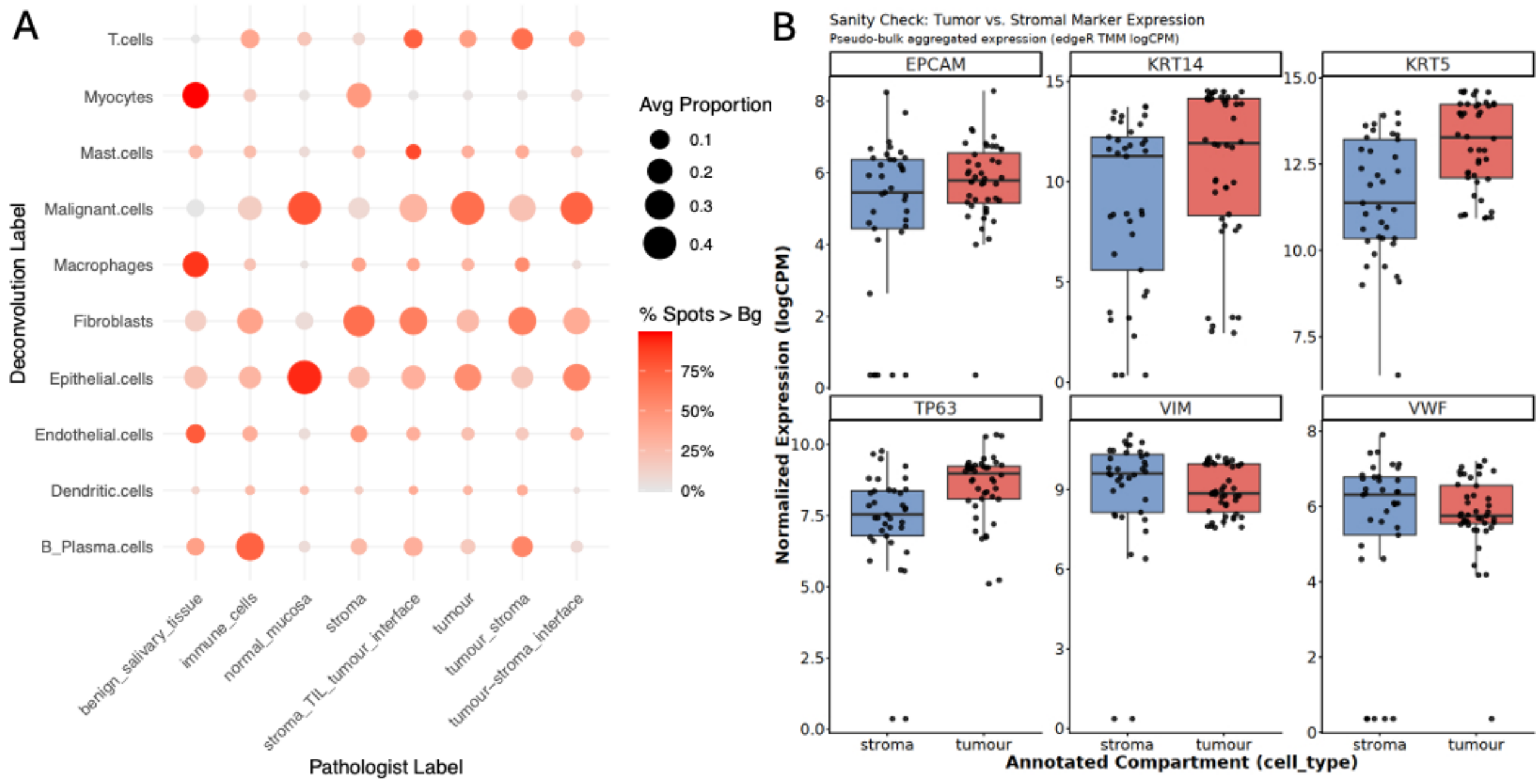

Figure S6. Validation of spatial compartmentalization and deconvolution. (A) Pathologist labels are shown along the x-axis (columns) versus deconvolution-derived cell types along the y-axis (rows). The area of each bubble corresponds to the mean estimated proportion of a cell type within the specific pathologist-labeled region. The bubble color intensity reflects the percentage of spots within that region exceeding the global background mean proportion for that cell type. **(B)** Sanity check of spatial annotations demonstrating the pseudo-bulk aggregated normalized expression (edgeR TMM logCPM) of canonical epithelial/tumor markers (*KRT5*, *KRT14*, *TP63*, *EPCAM*) and mesenchymal/stromal markers (*VIM*, *VWF*) across the annotated tumor and stroma compartments.

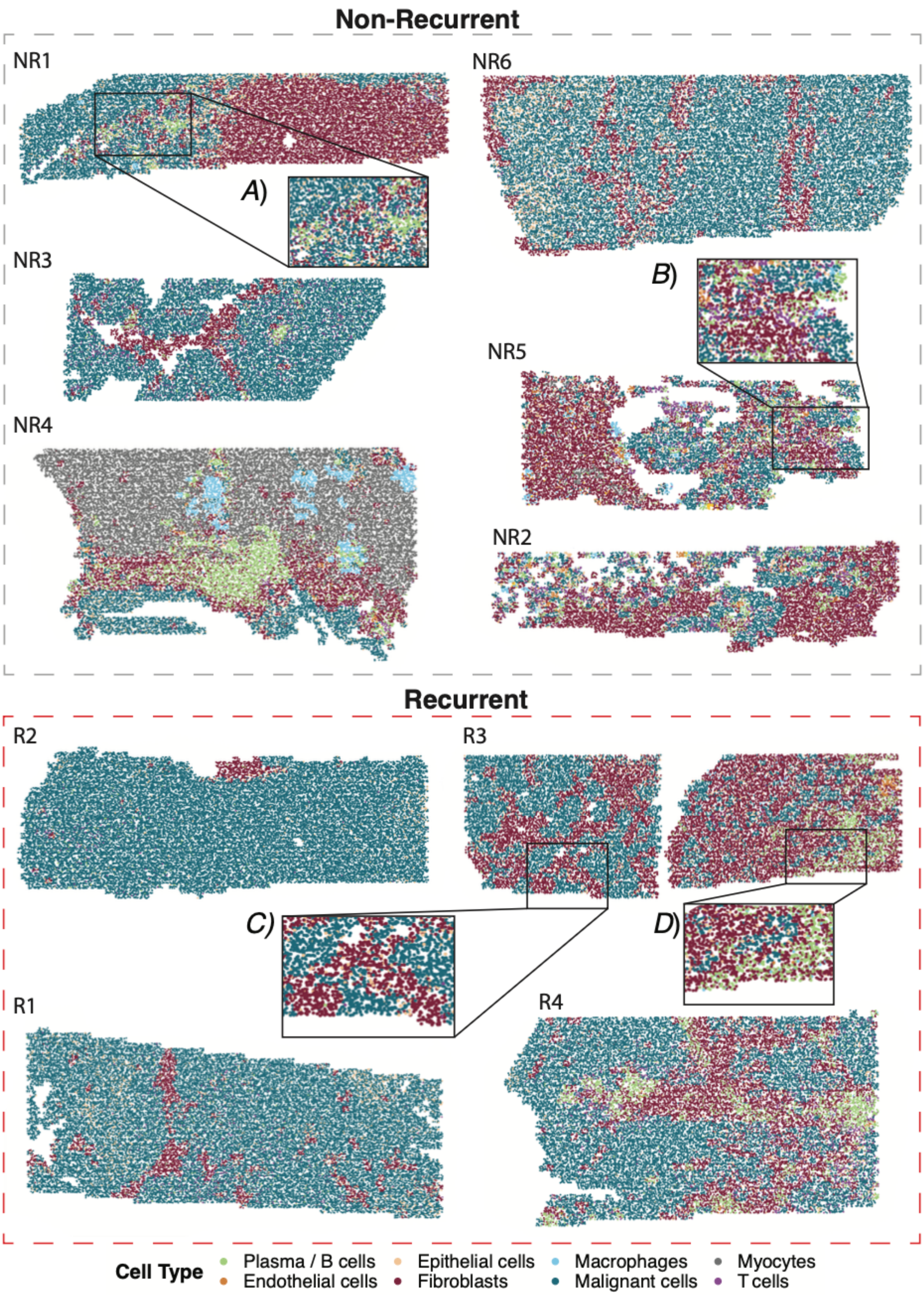
Non-Recurrent
NR1
A)
NR6
NR3
B)
NR5
NR4
NR2
Recurrent
R2
R3
C)
D)
R1
R4
Cell Type
Plasma / B cells
Endothelial cells
Epithelial cells
Fibroblasts
Macrophages
Malignant cells
Myocytes
T cells

Figure S7. CARD single-cell resolution mapping of deconvolution results. Spatial plots of inferred single-cell resolution for each sample. Sub-plots (A, B, C, D) represent regions of interest that highlight the colocalization of immune cells with tumor and stromal cells. NR = Non-recurrence, R = Recurrence.

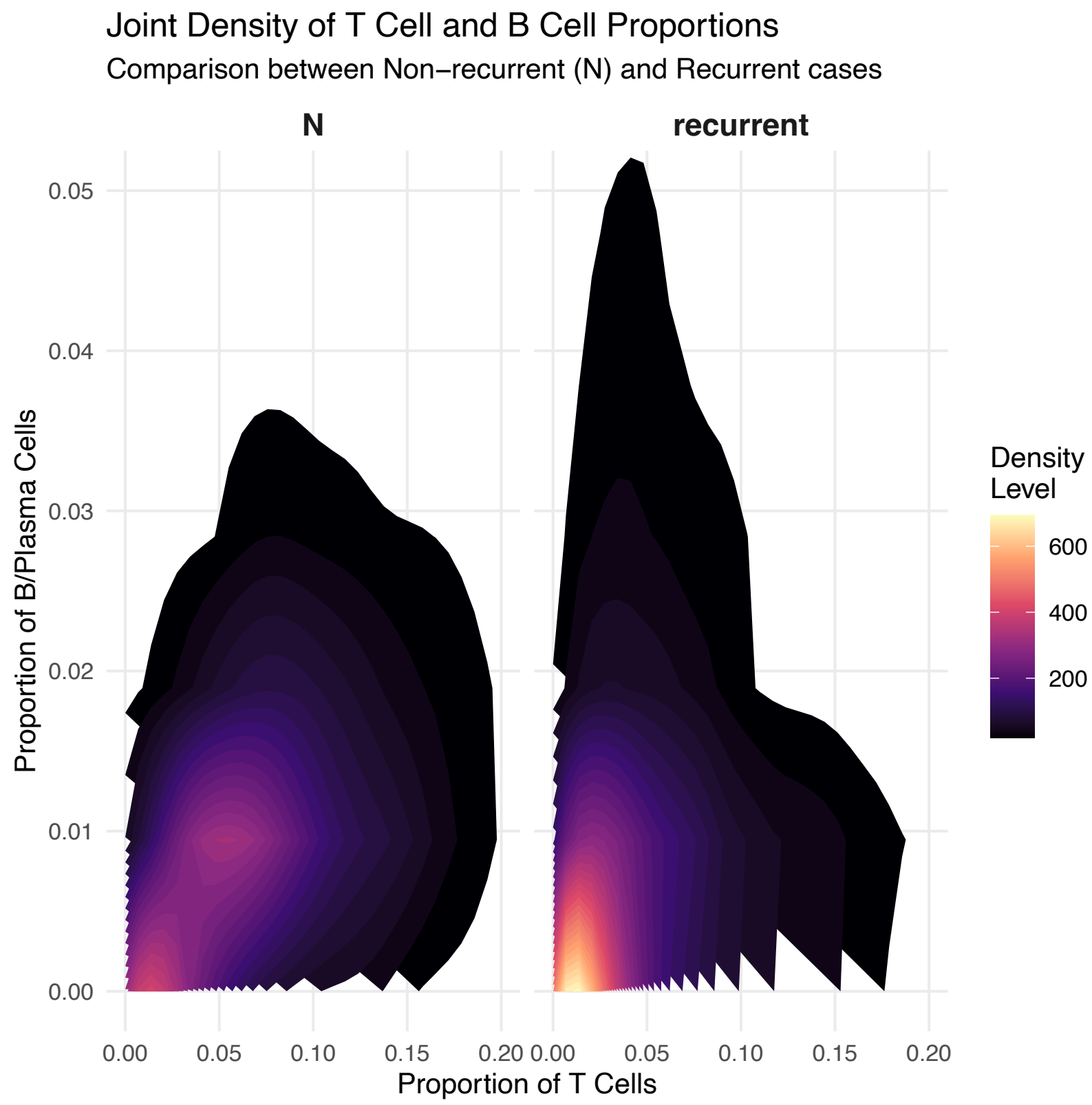

Figure S8. Quantitative density profiling of immune cell colocalization. Joint density plots comparing the proportions of T cells (x-axis) and B/plasma cells (y-axis) per Visium spot between non-recurrent (N) and recurrent tumors. The color gradient represents the density level of tumor spots. While recurrent samples are characterized by an immunologically quiescent phenotype (single peak at origin), non-recurrent samples exhibit a secondary population of spots showing higher joint infiltration of both lymphoid lineages.

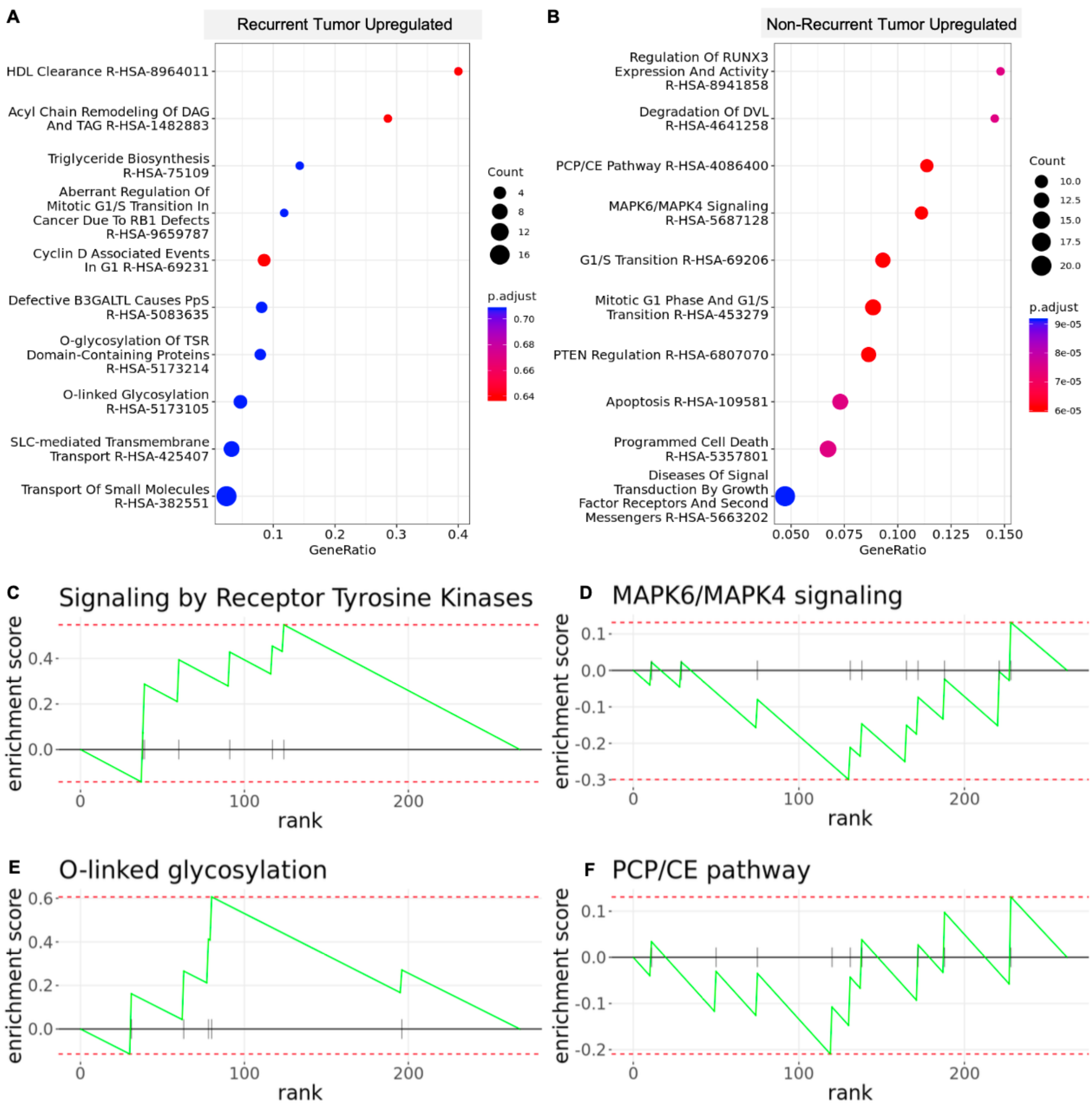

Figure S9. Pathway Analysis using ReactomeDB for differentially expressed genes between recurrent and non-recurrent tumors. (A-B) Top-10 GO terms associated with genes upregulated in recurrent (A) and non-recurrent (B) tumor samples. **(C-F)** GSEA analysis for the terms related to the genes upregulated in recurrent tumor samples (C, E) and non-recurrent tumor samples (D, F).

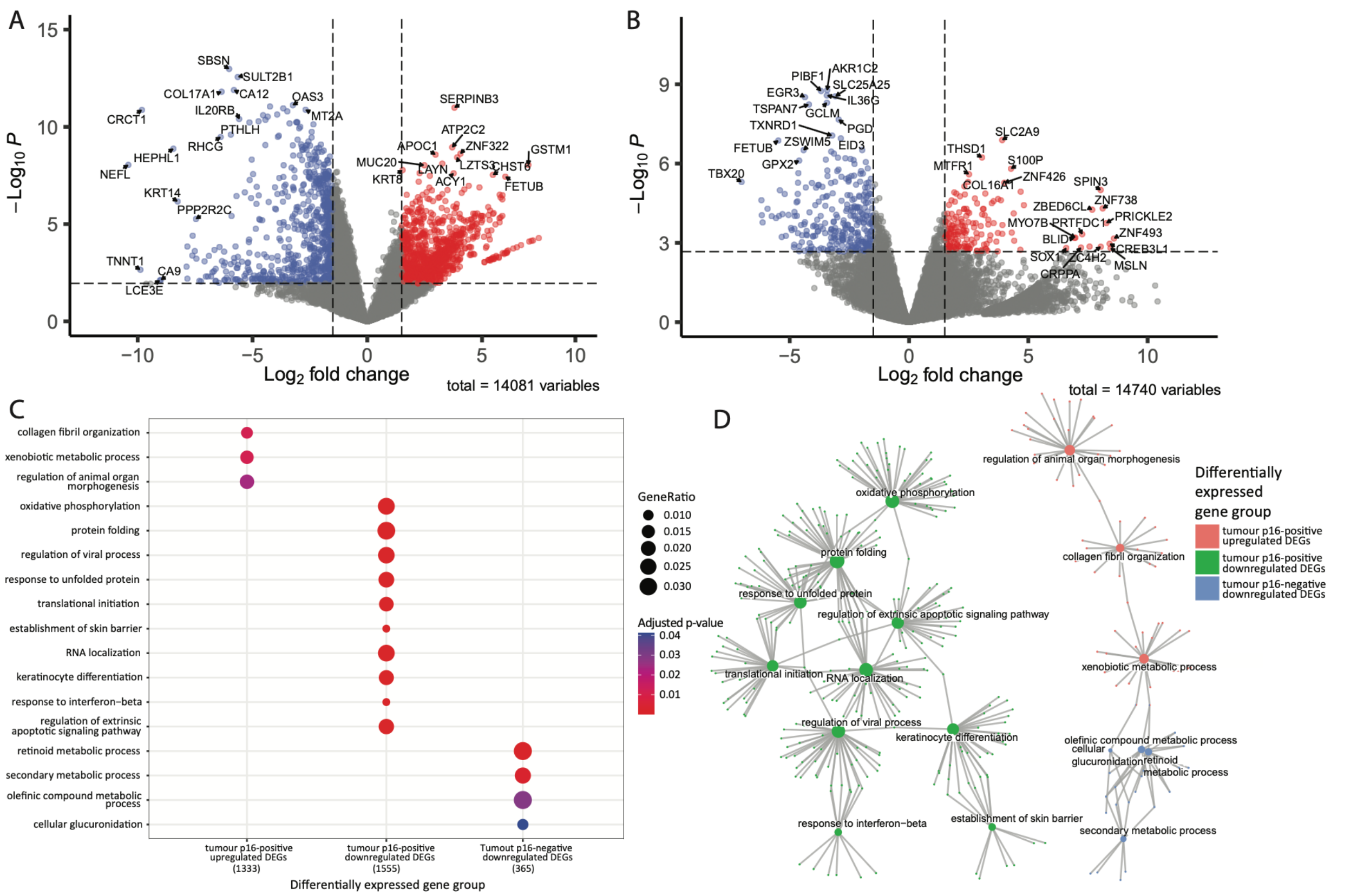

Figure S10. Differential gene expression analysis of recurrent tumor p16-positive and -negative samples and GO term analysis results. (A) Volcano plot of tumor p16-positive DE analysis with 1078 upregulated (log2FC > 1.5 and $P<1.12\times10^{-2}$) and 704 downregulated (log2FC < -1.5 and $P < 1.12\times10^{-2}$) genes highlighted in red and blue respectively. Some of the top genes are annotated. **(B)** Volcano plot of tumor p16-negative DE analysis with 172 upregulated (log2FC > 1.5 and $P< 2.14\times10^{-3}$) and 297 downregulated (log2FC < -1.5 and $P< 2.14\times10^{-3}$) genes highlighted in red and blue respectively. Some of the top genes are annotated. **(C)** Dot plot of top enriched GO terms for tumor p16-positive and negative DEGs. tumor p16-negative downregulated DEGs had no significantly enriched GO terms (BH-adjusted P<0.05). **(D)** Network plot of enriched GO terms for tumor p16-positive and negative DEGs. The connections depict the linkage of GO terms as a network. The core size represents the gene ratio of DEGs present in the GO term. Each spine represents a gene in the GO term that is present in the DEG list. Connections represent an overlap of the gene between GO terms.

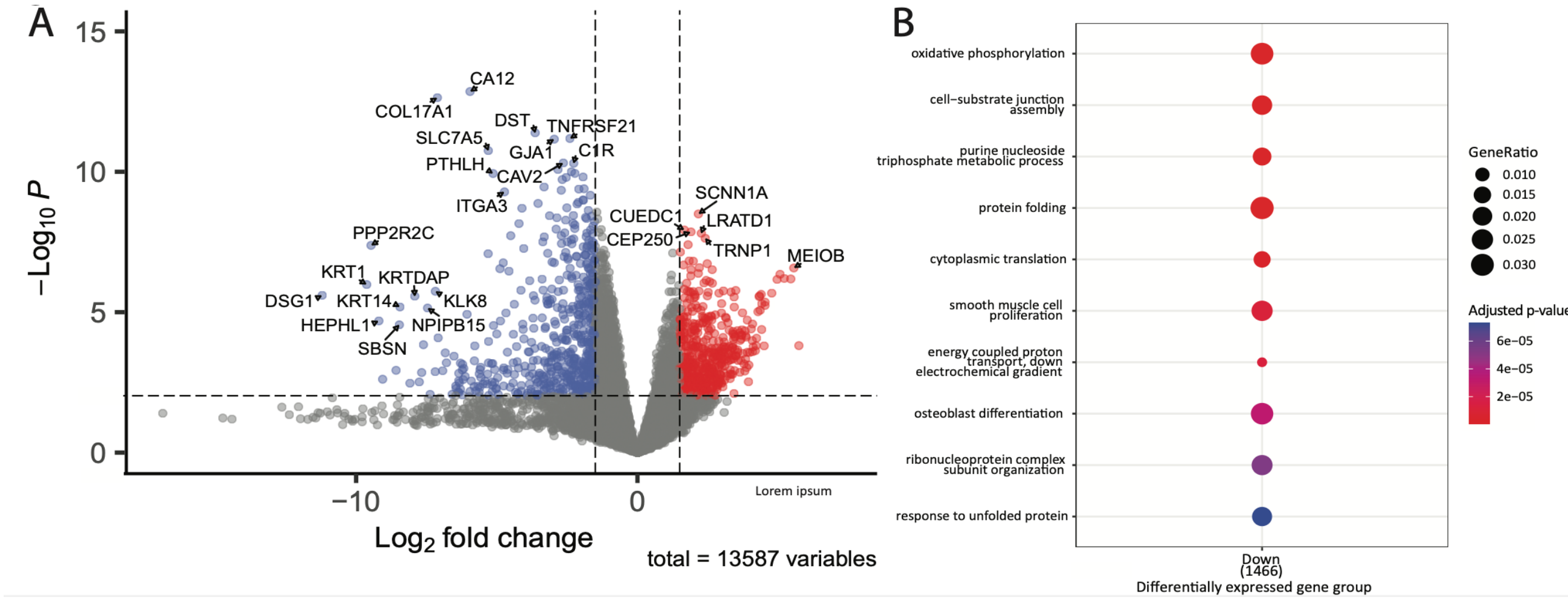

Figure S11. **Differential gene expression analysis of recurrent stroma samples and GO term analysis results**. **(A)** Volcano plot of recurrent stroma DE analysis with 523 upregulated (log2FC>1.5 and $P < 9.44\times10^{-3}$) and 566 downregulated (log2FC<-1.5 and $P < 9.44\times10^{-3}$) genes highlighted in red and blue respectively. Some of the top genes are annotated. **(B)** Dot plot of top 10 enriched GO terms for 1556 downregulated stromal DEGs (BH-adjusted P<0.05). Stromal upregulated DEGs (n=1012) showed no significantly enriched GO terms (BH-adjusted P<0.05).

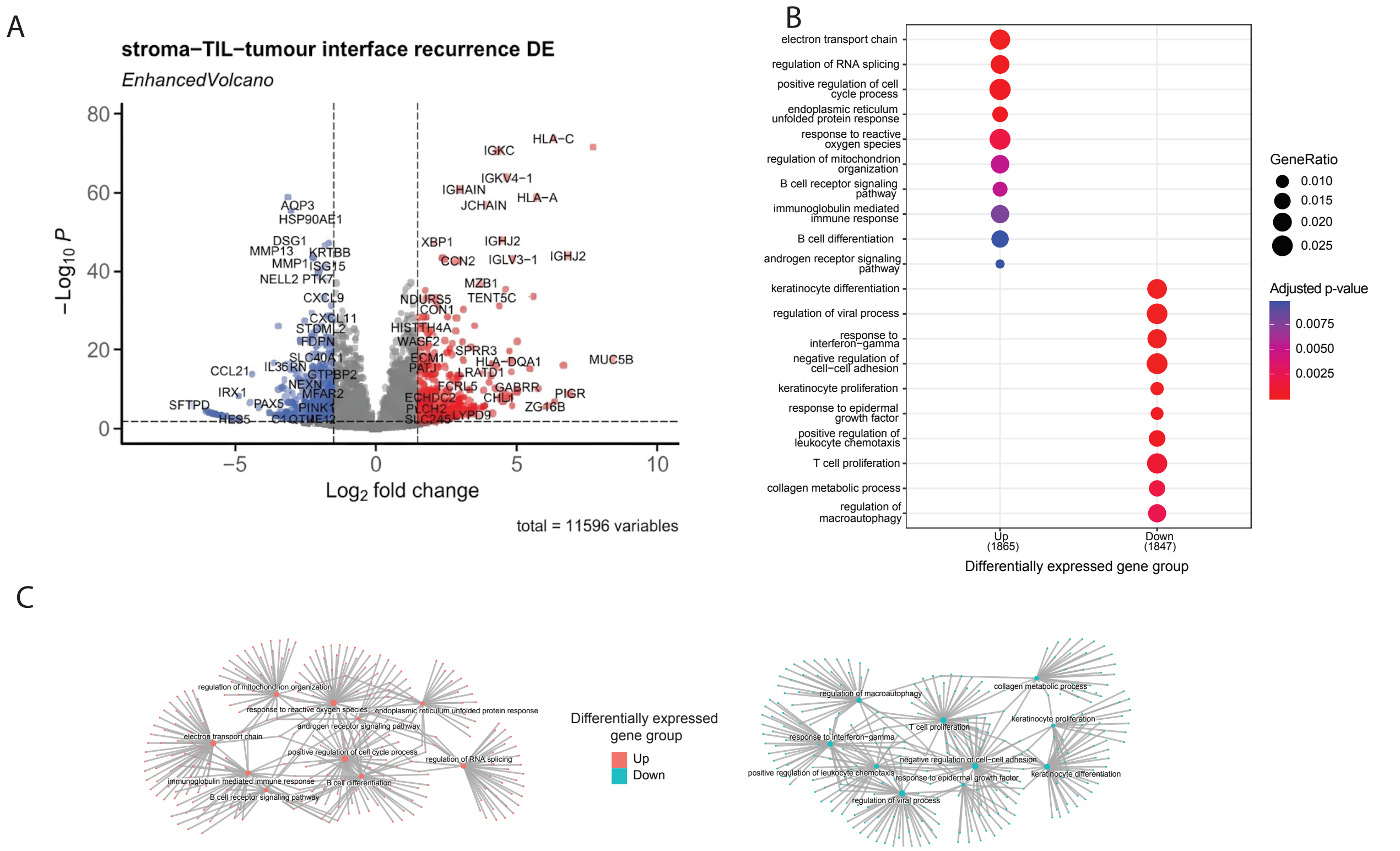

Figure S12. Differential gene expression analysis of stroma-TIL-tumor interfaces between recurrence and non-recurrence samples and GO term analysis results. (A) Volcano plot of recurrent stroma-TIL-tumor DE analysis with 412 upregulated (log2FC>1.5) and 541 downregulated (log2FC<-1.5) genes highlighted in red and blue respectively. Some of the top genes are annotated. **(B)** Dot plot of top 10 enriched GO terms for downregulated stroma-TIL-tumor DEGs (n=1847) and upregulated DEGs (n=1365) (BH-adjusted P<0.05). **(C)** Network plot shows the schematic connection of GO terms with colors of the nodes representing the upregulated (red) and downregulated (blue) DEG-associated GO terms. Connections represent an overlap of the gene between GO terms.

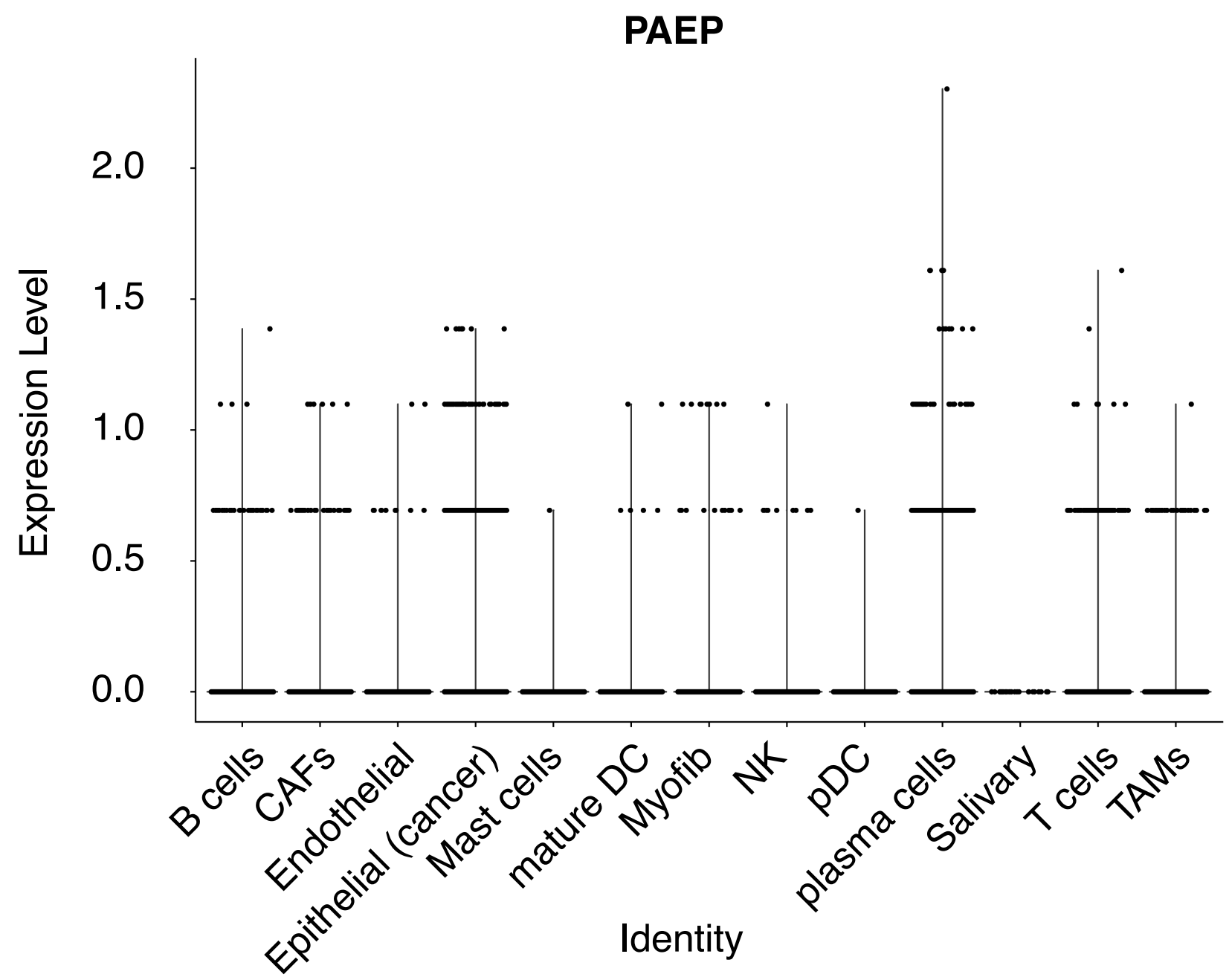

Figure S13. ***PAEP* expression profiles across cell types in HNC Xenium data.** The plot displays the distribution of *PAEP* expression levels across identified cell populations. Expression values represent Log-normalized transcript counts. Note that the discrete variation in signal intensity reflects the single-molecule nature of Xenium data, where low copy numbers result in distinct, non-continuous expression levels.

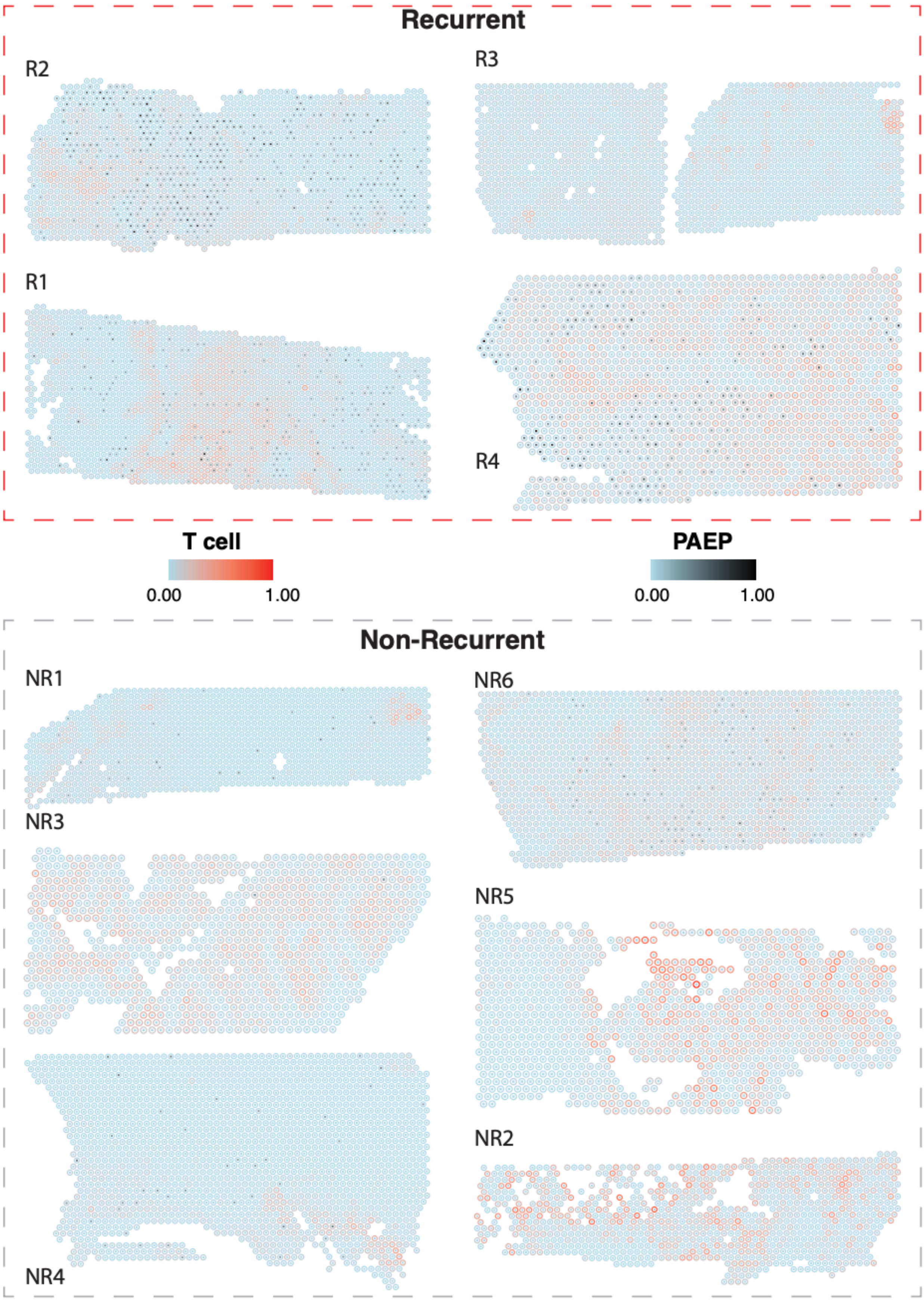
Recurrent
R2
R3
R1
R4
T cell
0.00
1.00
PAEP
0.00
1.00
Non-Recurrent
NR1
NR6
NR3
NR5
NR2
NR4

Figure S14. **Visualization of *PAEP* expression and T cell proportions in Visium spots for each sample**. For each spot, the edge color of the circle represents the relative abundance of T cells and plasma cells, while the center square color represents relative *PAEP* expression. Both values are scaled to the maximum across all samples (1 = highest expression or cell proportion). NR = Non-recurrent; R = Recurrent.

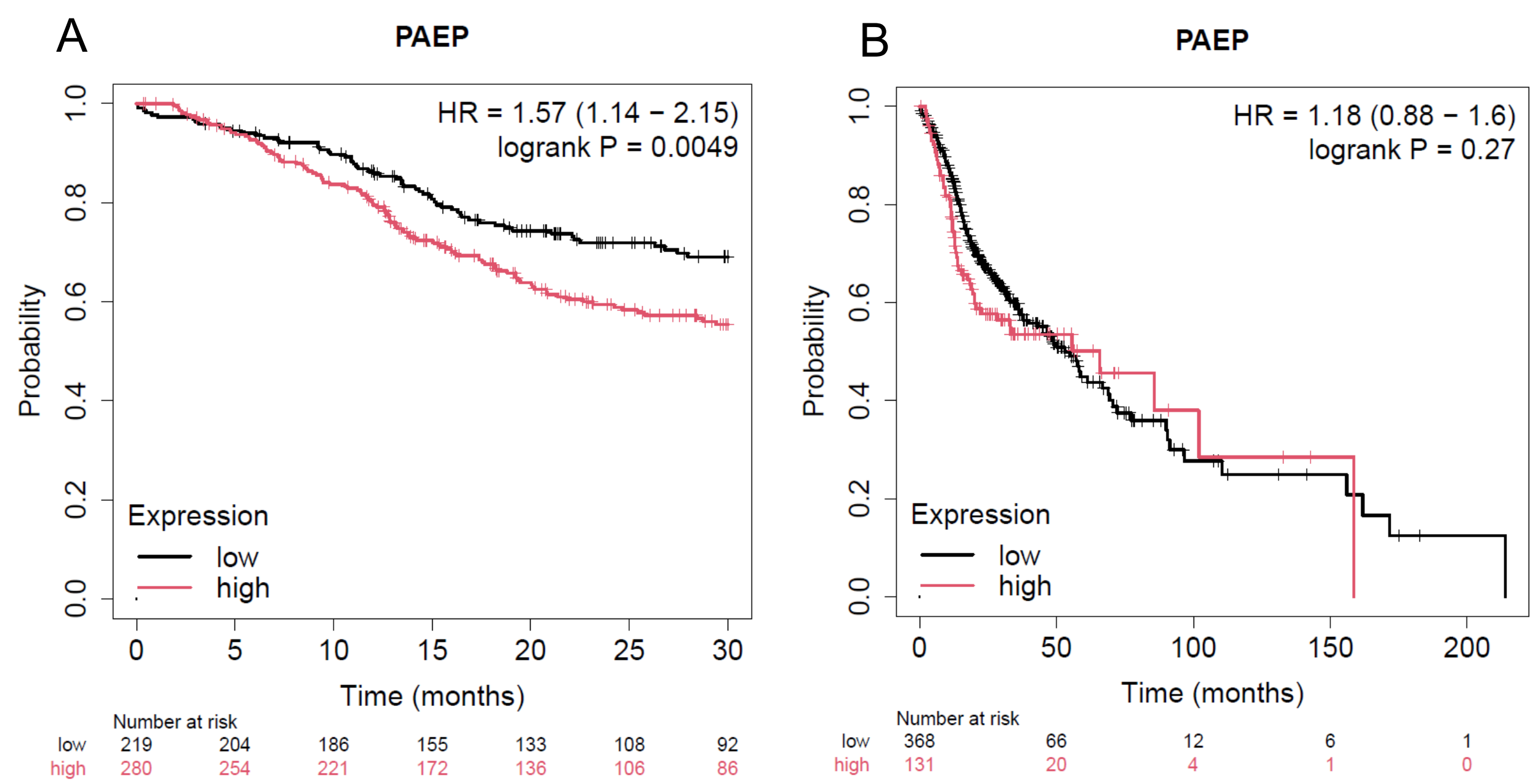

Figure S15. Kaplan Meier survival analysis of *PAEP* expression in an integrated public head and neck cancer cohort. (A) Kaplan Meier survival curve restricted to a 30 month follow up threshold to target the high risk recurrence window. High *PAEP* expression (red) is significantly associated with reduced overall survival compared to low expression (black) (HR = 1.57, 95% CI = 1.14 to 2.15, log rank P = 0.0049). (B) Kaplan Meier survival curve evaluating unconstrained long term overall survival up to 200 months, where the prognostic association is attenuated (HR = 1.18, 95% CI = 0.88 to 1.6, log rank P = 0.27). Corresponding number at risk tables are displayed below each chart.

2 Supplementary Tables

Table S1. Characteristics of patients used in this study.

**Recurrence status**	**Patient ID**	**Age**	**Sex**	**Cancer site**	**Anatomical region**	**Pathological stage (TNM staging system)**	**Cancer stage**	**P16 IHC**	**Vsium Slide**
Yes	R1	58	M	Left lateral tongue	Oral cavity	T4N1M0	4a	Negative	**B**
	R2	47	M	Left lateral tongue	Oral cavity	T3N2bM0	4a	Negative	**A**
	R3**	54	M	Left base of tongue	Oropharynx	T4aN2cM0	4a	Positive	**C**
	R4	75	M	Right floor of mouth	Oral cavity	T3N0M0	3	Negative	**D**
No	NR1	67	M	Left tonsil/soft palate	Oropharynx	T3N0M0	3	Negative	**A**
	NR2	64	F	Left oropharyngeal	Oropharynx	T2N1M0	3	Positive	**C**
	NR3	66	M	Left tonsil	Oropharynx	T2N2aM0	4a	Positive	**A**
	NR4	66	M	Right mandible	Jaw	T4N0M0	4a	Not Available	**B**
	NR5	55	M	Right oropharynx	Oropharynx	T1N1M0	3	Positive	**C**
	NR6	56	F	Left mandible	Jaw	T2N0M0	2	Not Available	**D**

** This patient had two tumour samples taken and embedded into two separate blocks which were subsequently sectioned and placed on the array independently. During DE analyses, the two tissues were considered as one sample.

Table S2. **TCGA samples used in this study.**

Cases submitter id	type	Demo-graphic age at index	Demo-graphic gender	Demo-graphic race	Age group	Exposures tobacco smoking status	Exposure alcohol history
**TCGA-BA-A6DL**	non-recurrence	59	male	white	40-59	Current Smoker	Yes
**TCGA-DQ-7592**	non-recurrence	57	male	american indian or alaska native	40-59	Current Smoker	Yes
**TCGA-MT-A67F**	non-recurrence	60	female	white	60-79	Lifelong Non-Smoker	No
**TCGA-HD-7832**	non-recurrence	52	male	white	40-59	Current Smoker	Yes
**TCGA-QK-A8Z7**	non-recurrence	59	male	black or african american	40-59	Current Smoker	No
**TCGA-CN-4740**	recurrence	79	female	white	60-79	Current Reformed Smoker for < or = 15 yrs	Yes
**TCGA-H7-8502**	non-recurrence	50	male	white	40-59	Current Smoker	Yes
**TCGA-CV-5966**	non-recurrence	63	female	white	60-79	Current Reformed Smoker for < or = 15 yrs	Yes
**TCGA-BA-5151**	recurrence	72	male	white	60-79	Current Reformed Smoker for > 15 yrs	No
**TCGA-DQ-5631**	recurrence	52	male	white	40-59	Current Reformed Smoker for < or = 15 yrs	Yes
**TCGA-CR-7386**	recurrence	69	male	white	60-79	Current Reformed Smoker for > 15 yrs	Yes
**TCGA-BA-5153**	recurrence	51	male	white	40-59	Lifelong Non-Smoker	No
**TCGA-CV-7235**	non-recurrence	67	male	white	60-79	Current Reformed Smoker for > 15 yrs	Yes
**TCGA-BA-5556**	non-recurrence	58	female	white	40-59	Current Reformed Smoker for < or = 15 yrs	Yes

**TCGA-HD-7754**	non-recurrence	69	male	white	60-79	Current Reformed Smoker for < or = 15 yrs	Yes
**TCGA-P3-A6T8**	non-recurrence	54	male	white	40-59	Current Smoker	Yes
**TCGA-CV-5444**	non-recurrence	64	male	black or african american	60-79	Current Reformed Smoker for < or = 15 yrs	Yes
**TCGA-CV-A6JN**	non-recurrence	53	male	white	40-59	Current Smoker	Yes
**TCGA-HD-7753**	non-recurrence	62	male	black or african american	60-79	Current Smoker	Yes
**TCGA-CX-7086**	non-recurrence	53	male	black or african american	40-59	Current Smoker	Yes
**TCGA-BA-5559**	recurrence	71	male	white	60-79	Lifelong Non-Smoker	No
**TCGA-F7-A50I**	non-recurrence	72	male	white	60-79	Current Smoker	Yes
**TCGA-BB-7864**	non-recurrence	61	male	white	60-79	Current Reformed Smoker for < or = 15 yrs	Yes
**TCGA-HD-8635**	non-recurrence	61	female	white	60-79	Current Smoker	Yes
**TCGA-CN-4736**	recurrence	70	female	white	60-79	Current Reformed Smoker for < or = 15 yrs	Yes
**TCGA-D6-6517**	recurrence	59	male	white	40-59	Current Reformed Smoker for < or = 15 yrs	Yes
**TCGA-CQ-A4C6**	non-recurrence	63	male	asian	60-79	Current Reformed Smoker for < or = 15 yrs	No
**TCGA-CR-6470**	non-recurrence	38	male	white	0-40	Lifelong Non-Smoker	Yes
**TCGA-QK-A8ZB**	non-recurrence	68	male	black or african american	60-79	Current Reformed Smoker for > 15 yrs	No
**TCGA-BA-5555**	recurrence	54	male	black or african american	40-59	Current Reformed Smoker for < or = 15 yrs	No
**TCGA-CR-6467**	non-recurrence	59	male	white	40-59	Current Reformed Smoker for < or = 15 yrs	Yes

**TCGA-CV-6933**	non-recurrence	53	male	white	40-59	Current Reformed Smoker for < or = 15 yrs	No
**TCGA-CV-6954**	non-recurrence	59	male	black or african american	40-59	Current Reformed Smoker for > 15 yrs	Yes
**TCGA-CQ-6221**	non-recurrence	79	male	white	60-79	Current Reformed Smoker for > 15 yrs	Yes
**TCGA-CV-7089**	non-recurrence	74	male	white	60-79	Current Reformed Smoker for < or = 15 yrs	No
**TCGA-IQ-A61E**	non-recurrence	55	female	white	40-59	Current Smoker	Yes
**TCGA-CR-7380**	recurrence	58	male	white	40-59	Lifelong Non-Smoker	No
**TCGA-HD-A6I0**	non-recurrence	56	male	white	40-59	Current Reformed Smoker for > 15 yrs	Yes
**TCGA-QK-A652**	non-recurrence	60	male	white	60-79	Lifelong Non-Smoker	Yes
**TCGA-CV-6003**	non-recurrence	50	female	white	40-59	Lifelong Non-Smoker	Yes
**TCGA-CN-A49A**	recurrence	60	male	white	60-79	Lifelong Non-Smoker	No
**TCGA-H7-8501**	non-recurrence	54	male	white	40-59	Current Smoker	Yes
**TCGA-CR-7390**	non-recurrence	67	male	white	60-79	Current Smoker	Yes
**TCGA-CV-A6JZ**	recurrence	68	male	white	60-79	Current Smoker	Yes
**TCGA-BA-6869**	non-recurrence	62	male	white	60-79	Current Smoker	Yes
**TCGA-CV-A6K0**	recurrence	58	male	white	40-59	Lifelong Non-Smoker	Yes
**TCGA-CN-A63T**	non-recurrence	60	male	white	60-79	Current Smoker	Yes
**TCGA-BB-8596**	non-recurrence	69	female	white	60-79	Current Reformed Smoker for < or = 15 yrs	Yes
**TCGA-CX-A4AQ**	non-recurrence	56	male	white	40-59	Current Reformed Smoker for < or = 15 yrs	Yes
**TCGA-CQ-6219**	recurrence	50	female	white	40-59	Current Smoker	Yes

**TCGA-F7-A61W**	non-recurrence	51	male	asian	40-59	Current Smoker	Yes
**TCGA-CV-6950**	recurrence	64	male	white	60-79	Current Reformed Smoker for < or = 15 yrs	Yes
**TCGA-CV-6441**	recurrence	60	male	white	60-79	Current Reformed Smoker for < or = 15 yrs	Yes
**TCGA-BB-A5HZ**	recurrence	65	male	white	60-79	Lifelong Non-Smoker	No
**TCGA-MT-A7BN**	recurrence	74	male	white	60-79	Lifelong Non-Smoker	No
**TCGA-CX-7085**	recurrence	77	female	white	60-79	Current Reformed Smoker for > 15 yrs	No
**TCGA-BA-5149**	recurrence	47	male	white	40-59	Current Smoker	Yes
**TCGA-CR-7397**	non-recurrence	44	male	white	40-59	Current Reformed Smoker for < or = 15 yrs	Yes
**TCGA-D6-8568**	non-recurrence	62	male	white	60-79	Current Smoker	Yes
**TCGA-T2-A6X2**	non-recurrence	82	male	white	80+	Current Reformed Smoker for > 15 yrs	No
**TCGA-CN-6019**	non-recurrence	61	male	white	60-79	Current Reformed Smoker for < or = 15 yrs	Yes
**TCGA-BA-7269**	non-recurrence	61	male	white	60-79	Current Smoker	Yes
**TCGA-CR-5243**	non-recurrence	51	male	white	40-59	Lifelong Non-Smoker	No
**TCGA-MZ-A6I9**	recurrence	68	male	white	60-79	Current Reformed Smoker for > 15 yrs	Yes
**TCGA-CQ-A4CH**	recurrence	58	male	asian	40-59	Lifelong Non-Smoker	No
**TCGA-QK-A64Z**	recurrence	79	female	white	60-79	Lifelong Non-Smoker	No
**TCGA-CN-6989**	recurrence	64	male	white	60-79	Current Smoker	Yes
**TCGA-BB-4227**	recurrence	66	male	white	60-79	Current Reformed Smoker for > 15 yrs	Yes
**TCGA-BA-A4IH**	non-recurrence	57	male	white	40-59	Current Reformed	Yes

						Smoker for < or = 15 yrs	
**TCGA-KU-A6H7**	non-recurrence	55	female	white	40-59	Current Reformed Smoker for < or = 15 yrs	Yes
**TCGA-CV-7418**	recurrence	62	male	black or african american	60-79	Current Reformed Smoker for < or = 15 yrs	Yes
**TCGA-DQ-7589**	non-recurrence	70	male	white	60-79	Current Reformed Smoker for < or = 15 yrs	Yes
**TCGA-CV-A6K1**	non-recurrence	65	male	white	60-79	Current Reformed Smoker for < or = 15 yrs	Yes
**TCGA-CV-5441**	non-recurrence	58	male	white	40-59	Current Reformed Smoker for < or = 15 yrs	Yes
**TCGA-DQ-7594**	non-recurrence	47	male	white	40-59	Lifelong Non-Smoker	Yes
**TCGA-HD-8634**	non-recurrence	51	female	white	40-59	Current Smoker	Yes
**TCGA-H7-A76A**	non-recurrence	57	male	white	40-59	Current Reformed Smoker for < or = 15 yrs	Yes
**TCGA-CV-A6JU**	non-recurrence	61	female	white	60-79	Current Smoker	Yes
**TCGA-CV-6959**	recurrence	48	male	white	40-59	Lifelong Non-Smoker	Yes
**TCGA-F7-A61S**	recurrence	62	male	white	60-79	Current Reformed Smoker for < or = 15 yrs	Yes
**TCGA-BB-7863**	non-recurrence	43	female	white	40-59	Lifelong Non-Smoker	No
**TCGA-MZ-A7D7**	recurrence	51	male	white	40-59	Current Smoker	Yes
**TCGA-CN-A499**	non-recurrence	60	female	white	60-79	Lifelong Non-Smoker	No
**TCGA-BB-8601**	recurrence	84	male	black or african american	80+	Current Reformed Smoker for < or = 15 yrs	No
**TCGA-F7-A50J**	non-recurrence	67	female	white	60-79	Current Reformed Smoker for < or = 15 yrs	Yes

**TCGA-BA-A6DI**	recurrence	62	male	black or african american	60-79	Current Smoker	Yes
**TCGA-D6-6823**	non-recurrence	50	male	white	40-59	Current Smoker	Yes
**TCGA-CN-5358**	recurrence	60	male	white	60-79	Current Reformed Smoker for < or = 15 yrs	Yes
**TCGA-F7-A623**	non-recurrence	70	male	white	60-79	Current Smoker	Yes
**TCGA-CQ-7069**	non-recurrence	77	female	white	60-79	Lifelong Non-Smoker	No
**TCGA-D6-6825**	non-recurrence	73	male	white	60-79	Current Reformed Smoker for < or = 15 yrs	Yes
**TCGA-KU-A6H8**	recurrence	41	male	white	40-59	Current Smoker	Yes
**TCGA-CN-A6V6**	non-recurrence	59	male	white	40-59	Current Reformed Smoker for < or = 15 yrs	Yes
**TCGA-DQ-5625**	recurrence	52	female	white	40-59	Current Smoker	Yes
**TCGA-CV-5977**	non-recurrence	66	male	white	60-79	Current Reformed Smoker for < or = 15 yrs	Yes
**TCGA-CR-7385**	non-recurrence	42	male	white	40-59	Current Reformed Smoker for > 15 yrs	Yes
**TCGA-CQ-7071**	non-recurrence	76	female	white	60-79	Lifelong Non-Smoker	No
**TCGA-T2-A6X0**	non-recurrence	49	male	white	40-59	Current Reformed Smoker for < or = 15 yrs	No
**TCGA-CR-7367**	non-recurrence	52	male	white	40-59	Current Smoker	Yes
**TCGA-CN-6016**	non-recurrence	64	male	white	60-79	Current Smoker	Yes
**TCGA-F7-7848**	non-recurrence	47	male	white	40-59	Current Smoker	Yes
**TCGA-CN-6017**	non-recurrence	55	male	white	40-59	Current Reformed Smoker for > 15 yrs	Yes
**TCGA-MT-A51W**	non-recurrence	52	female	white	40-59	Current Smoker	Yes
**TCGA-MT-A67A**	non-recurrence	85	female	white	80+	Lifelong Non-Smoker	No

**TCGA-CV-A6JE**	non-recurrence	78	male	white	60-79	Current Reformed Smoker for < or = 15 yrs	Yes
**TCGA-TN-A7HL**	non-recurrence	59	male	white	40-59	Current Reformed Smoker for < or = 15 yrs	Yes
**TCGA-CN-A63W**	non-recurrence	48	female	white	40-59	Current Reformed Smoker for < or = 15 yrs	No
**TCGA-CV-6936**	recurrence	68	male	white	60-79	Current Smoker	No
**TCGA-DQ-5630**	non-recurrence	73	male	white	60-79	Current Reformed Smoker for > 15 yrs	Yes
**TCGA-CQ-A4CG**	recurrence	78	female	white	60-79	Current Reformed Smoker for < or = 15 yrs	No
**TCGA-CV-5440**	non-recurrence	52	male	white	40-59	Current Smoker	Yes
**TCGA-BB-4224**	recurrence	52	male	white	40-59	Lifelong Non-Smoker	Yes
**TCGA-H7-7774**	non-recurrence	75	female	white	60-79	Current Reformed Smoker for < or = 15 yrs	No
**TCGA-CV-7434**	non-recurrence	64	male	white	60-79	Current Reformed Smoker for < or = 15 yrs	Yes
**TCGA-CR-5249**	non-recurrence	35	female	white	0-40	Lifelong Non-Smoker	Yes
**TCGA-CQ-7063**	recurrence	59	female	white	40-59	Current Reformed Smoker for > 15 yrs	No
**TCGA-CN-A641**	non-recurrence	47	male	white	40-59	Current Smoker	No
**TCGA-QK-A6IF**	recurrence	61	male	white	60-79	Lifelong Non-Smoker	Yes
**TCGA-DQ-7596**	recurrence	48	male	white	40-59	Lifelong Non-Smoker	Yes
**TCGA-T2-A6WZ**	recurrence	53	male	white	40-59	Current Smoker	Yes
**TCGA-HD-8224**	recurrence	63	male	white	60-79	Lifelong Non-Smoker	Yes
**TCGA-CQ-5323**	non-recurrence	82	male	white	80+	Current Reformed	Yes

						Smoker for > 15 yrs	
**TCGA-QK-A6II**	recurrence	52	male	white	40-59	Current Smoker	Yes
**TCGA-CQ-A4CD**	non-recurrence	69	male	white	60-79	Lifelong Non-Smoker	No
**TCGA-F7-A624**	non-recurrence	73	male	white	60-79	Lifelong Non-Smoker	Yes
**TCGA-HD-7831**	recurrence	74	male	white	60-79	Lifelong Non-Smoker	Yes
**TCGA-CV-7427**	recurrence	73	female	white	60-79	Current Reformed Smoker for > 15 yrs	Yes
**TCGA-CV-5431**	non-recurrence	73	male	white	60-79	Current Reformed Smoker for < or = 15 yrs	Yes
**TCGA-CN-4728**	non-recurrence	56	male	white	40-59	Current Smoker	Yes
**TCGA-UP-A6WW**	non-recurrence	58	male	white	40-59	Lifelong Non-Smoker	Yes
**TCGA-CQ-A4CI**	recurrence	73	male	white	60-79	Current Reformed Smoker for < or = 15 yrs	No
**TCGA-CV-6960**	non-recurrence	49	male	black or african american	40-59	Current Reformed Smoker for < or = 15 yrs	Yes
**TCGA-CQ-6224**	non-recurrence	52	male	white	40-59	Current Reformed Smoker for < or = 15 yrs	Yes
**TCGA-CV-A6K2**	recurrence	79	male	white	60-79	Current Reformed Smoker for > 15 yrs	Yes
**TCGA-DQ-7588**	recurrence	65	male	white	60-79	Current Reformed Smoker for < or = 15 yrs	Yes
**TCGA-CV-6952**	non-recurrence	65	female	white	60-79	Current Reformed Smoker for < or = 15 yrs	Yes
**TCGA-F7-A622**	recurrence	75	male	white	60-79	Lifelong Non-Smoker	Yes
**TCGA-CN-6988**	non-recurrence	47	male	white	40-59	Current Reformed Smoker for < or = 15 yrs	Yes

**TCGA-BA-A6DD**	recurrence	44	male	black or african american	40-59	Current Smoker	Yes
**TCGA-P3-A6T2**	non-recurrence	45	male	white	40-59	Lifelong Non-Smoker	Yes
**TCGA-CN-5366**	recurrence	51	male	white	40-59	Current Smoker	Yes
**TCGA-CN-5355**	non-recurrence	64	male	black or african american	60-79	Current Smoker	Yes
**TCGA-BB-7871**	recurrence	64	female	white	60-79	Current Smoker	No
**TCGA-DQ-7595**	non-recurrence	53	male	white	40-59	Current Smoker	Yes
**TCGA-H7-A6C4**	recurrence	35	female	white	0-40	Current Smoker	No
**TCGA-BA-A6DB**	recurrence	24	female	white	0-40	Lifelong Non-Smoker	No
**TCGA-CV-7101**	non-recurrence	80	male	white	80+	Current Smoker	Yes
**TCGA-BA-5558**	non-recurrence	65	male	white	60-79	Lifelong Non-Smoker	No
**TCGA-CN-4729**	non-recurrence	73	male	white	60-79	Current Reformed Smoker for < or = 15 yrs	Yes
**TCGA-BA-A6DJ**	recurrence	62	male	asian	60-79	Current Reformed Smoker for < or = 15 yrs	No
**TCGA-CQ-A4C7**	recurrence	88	male	white	80+	Current Reformed Smoker for > 15 yrs	Yes
**TCGA-D6-6824**	non-recurrence	61	male	white	60-79	Current Smoker	Yes
**TCGA-CN-6010**	non-recurrence	53	male	white	40-59	Current Smoker	Yes
**TCGA-BB-7870**	non-recurrence	58	male	white	40-59	Current Smoker	Yes
**TCGA-DQ-7593**	non-recurrence	58	male	white	40-59	Current Smoker	Yes
**TCGA-CN-A49B**	non-recurrence	71	male	white	60-79	Current Reformed Smoker for < or = 15 yrs	Yes
**TCGA-P3-A6SW**	non-recurrence	50	male	white	40-59	Current Smoker	Yes
**TCGA-BA-6873**	recurrence	28	male	white	0-40	Current Smoker	No
**TCGA-CN-4735**	non-recurrence	52	male	white	40-59	Current Smoker	Yes

**TCGA-CV-7091**	non-recurrence	54	male	white	40-59	Current Reformed Smoker for < or = 15 yrs	Yes
**TCGA-DQ-7590**	non-recurrence	51	male	white	40-59	Current Reformed Smoker for > 15 yrs	Yes
**TCGA-CV-7243**	non-recurrence	50	male	white	40-59	Lifelong Non-Smoker	Yes
**TCGA-CQ-5325**	recurrence	65	male	white	60-79	Current Reformed Smoker for < or = 15 yrs	Yes
**TCGA-DQ-7591**	non-recurrence	62	male	white	60-79	Current Reformed Smoker for > 15 yrs	Yes
**TCGA-CV-7242**	non-recurrence	60	female	white	60-79	Current Reformed Smoker for > 15 yrs	Yes
**TCGA-BA-A6DE**	non-recurrence	70	female	white	60-79	Current Smoker	Yes
**TCGA-F7-8298**	non-recurrence	58	male	white	40-59	Current Reformed Smoker for > 15 yrs	Yes
**TCGA-BA-5152**	non-recurrence	56	male	white	40-59	Lifelong Non-Smoker	No
**TCGA-UF-A7J9**	non-recurrence	75	male	white	60-79	Current Smoker	Yes
**TCGA-CX-7082**	non-recurrence	82	male	white	80+	Current Reformed Smoker for < or = 15 yrs	No
**TCGA-RS-A6TP**	non-recurrence	58	male	white	40-59	Current Reformed Smoker for < or = 15 yrs	No
**TCGA-CR-7389**	non-recurrence	55	male	black or african american	40-59	Current Smoker	Yes
**TCGA-CV-5435**	recurrence	57	male	white	40-59	Current Reformed Smoker for < or = 15 yrs	Yes
**TCGA-QK-A8ZA**	non-recurrence	60	male	black or african american	60-79	Current Smoker	Yes
**TCGA-P3-A6T6**	recurrence	53	male	white	40-59	Lifelong Non-Smoker	Yes

**TCGA-BA-A8YP**	non-recurrence	50	male	black or african american	40-59	Current Smoker	Yes

Table S3. Differentially expressed genes between the tumor regions of recurrent and non-recurrent samples.

genes	logFC	logCPM	F	PValue
CWH43	3.97485694	4.70919727	25.7861509	2.31E-05
FMO2	3.77929733	4.02497861	17.7241523	0.00024296
CASP14	3.76810382	6.73876128	10.914841	0.00273324
GDPD2	3.57368575	3.23752696	17.3234529	0.00025626
BPIFB1	3.50834969	4.12092561	8.4817851	0.00717581
PAEP	3.4607129	3.9775054	16.7834897	0.00032842
TREX2	3.41683077	3.63732585	18.5792085	0.00017499
MARCO	3.35383244	3.14636275	21.8234561	5.98E-05
HS3ST6	3.30976087	4.96514763	9.51889557	0.00471112
PNPLA3	3.17699374	3.20785493	39.3459275	6.48E-07
ZNF878	3.12073079	1.80725207	19.1893334	0.00013262
IFNE	3.11330266	3.62411565	21.3660991	7.03E-05
OTOP3	3.05294527	3.66036944	9.62101972	0.00439247
MAGEA4	3.0527737	5.46655647	35.0555694	2.08E-06
PLAG1	2.94635212	2.91949088	34.6275442	1.92E-06
EFR3B	2.91585565	2.65402095	15.1084944	0.00051959
SMIM22	2.89685688	3.50973014	10.7288753	0.00279973
LDHD	2.89161455	2.790828	33.9478659	2.25E-06
TFF3	2.89119793	2.88229401	10.3401195	0.0031892
PRR9	2.87040205	6.15766426	6.34859487	0.01808324
CRNN	2.85795643	6.34505529	7.00899255	0.01347027
SLC7A4	2.79467246	1.4840923	22.5710783	4.69E-05
WDR72	2.7941572	4.57331121	19.2341751	0.0001467
KRBOX1	2.79094258	1.78715478	17.4882751	0.00023059
ATP2C2	2.78108105	5.30112094	37.988243	9.62E-07
PHYHD1	2.7678651	4.34881443	21.3374358	7.33E-05
PNMA5	2.76573229	5.27366221	5.33468365	0.02892746
FEZ1	2.72692364	6.34479327	8.89322871	0.0060654
ALPL	2.69866928	5.42329209	9.3103834	0.00512181
TFPI2	2.67407816	2.84995204	12.3448308	0.0014354
CHP2	2.66992342	1.93626535	12.6162618	0.0012844
CEL	2.62772505	4.46988733	8.90310329	0.0059973
PCDHB2	2.62388869	1.51605272	24.3430712	2.80E-05
SLC15A1	2.61851942	1.37359321	23.9374058	3.14E-05
CNTN1	2.60011024	4.50832661	38.6326297	7.60E-07
GPR78	2.59893927	2.74539206	10.2913244	0.00323123
CHRM3	2.59173309	1.99264969	12.2304361	0.00148584
DMBT1	2.57281368	2.34486681	6.36415007	0.0173874
LRG1	2.55529106	3.71926758	40.1509642	5.43E-07

**OR5P3**	2.55472089	1.79500493	20.1313667	9.85E-05
**PRR4**	2.55262913	3.37360181	9.35880413	0.00478231
**SLC15A2**	2.53248409	3.83256173	14.3364472	0.00071039
**MOXD1**	2.4885523	2.32825119	15.2124741	0.0005009
**CES3**	2.48821575	3.63560345	8.94028284	0.0057464
**KCP**	2.4708135	3.20506377	31.4361903	4.18E-06
**PIGR**	2.45445756	2.4137205	21.5201517	6.43E-05
**GSTM1**	2.4521702	3.66396666	7.87689312	0.00906031
**AJAP1**	2.43383776	4.76609562	8.3409886	0.00759143
**SLC47A2**	2.4288657	3.99243218	8.6297177	0.00658811
**ADAMTSL4**	2.42808525	4.87563751	23.0308991	4.41E-05
**REEP6**	2.41026502	3.21669392	26.8006196	1.41E-05
**KRT23**	2.40718111	5.93964766	12.545343	0.00146388
**ZNF433**	2.362852	2.7242487	19.9145566	0.00010543
**FAM3D**	2.3565813	5.14395973	8.18119699	0.00811678
**ZNF695**	2.35315711	2.52719818	13.0202755	0.00110452
**ZNF322**	2.3394368	5.61946058	19.8158323	0.00012278
**ACP7**	2.3373823	4.03062942	7.4990946	0.01070152
**HMGA2**	2.3294395	6.66318058	6.14042118	0.01987815
**DNAH14**	2.32893702	2.04569071	13.6521924	0.00087524
**PLAT**	2.32765101	4.86923171	23.867816	3.39E-05
**ATP6V1C2**	2.32123938	3.89364918	13.2553175	0.00104824
**FAM83E**	2.3158638	4.14916613	8.53941963	0.00684481
**USH1G**	2.31269461	2.6337725	11.2458151	0.00217091
**ACY1**	2.30250738	4.37656464	42.340976	3.39E-07
**CDKN1C**	2.28279006	4.41846272	21.7411015	6.14E-05
**LOR**	2.28206159	2.50835242	7.82560965	0.00899222
**TMC5**	2.27665887	2.48609263	13.6538677	0.0008747
**CSF3**	2.24533434	3.66610108	7.9935843	0.00855738
**CXCR2**	2.23117536	1.58302988	18.3869484	0.0001717
**CDSN**	2.22652671	6.78984617	4.4158309	0.0452409
**RERG**	2.21814416	2.21307612	9.90087655	0.0037116
**GLDC**	2.20390677	2.69743126	9.05946413	0.00527376
**SLC6A15**	2.20062204	3.47473503	4.3635211	0.0460287
**LGI3**	2.18564273	4.31575813	6.20964534	0.01917347
**FIGN**	2.18338809	2.21197471	15.9344961	0.00038978
**SPTSSB**	2.17807493	2.31203147	8.9424804	0.00551706
**AGR2**	2.17021498	2.22459013	14.7133762	0.00059734
**PLA2G4D**	2.16773892	3.48333286	5.31812227	0.02880803
**SCNN1D**	2.15643168	4.84755872	10.7552135	0.00280646
**SMOC1**	2.15497421	2.79996096	9.06887332	0.00525897
**NPR3**	2.1536967	3.66946257	7.59717241	0.01011402

**SERPINA3**	2.13590018	3.40447581	6.45854061	0.01684407
**MME**	2.13409589	4.05683544	11.3775257	0.00214435
**BARX2**	2.13005775	3.00656231	13.9835125	0.00077594
**BLID**	2.12945479	1.90040438	12.6975814	0.00124581
**JPH2**	2.12911787	3.75351296	6.51824154	0.01645314
**HYAL1**	2.12785332	5.15009508	28.7970151	8.32E-06
**CP**	2.12317686	2.95690543	9.35132851	0.00467268
**SHF**	2.10493969	2.71991588	9.81669014	0.00384122
**LZTS3**	2.10359819	4.53470986	13.2282611	0.00107544
**IGSF11**	2.10347602	2.83413653	6.05041461	0.020102
**ANKRD65**	2.10050773	3.97794048	11.8537342	0.0017662
**C4orf19**	2.09214053	3.13865854	6.36222574	0.01744369
**ZNF391**	2.08439391	1.99256651	21.464171	6.54E-05
**SLC6A14**	2.07988473	4.69283398	17.9028603	0.00021002
**PLA2G4A**	2.05361615	2.72944593	13.0654897	0.00108614
**SH3TC2**	2.04488608	2.17494643	9.8189419	0.00383769
**IL36B**	2.0428192	2.19240461	7.5956158	0.0098618
**TNFRSF6B**	2.04123355	6.25000455	22.210874	5.63E-05
**MC1R**	2.04059862	2.13899841	16.2308301	0.00035211
**NLGN4X**	2.03545898	2.55020442	9.69963926	0.0040296
**HTR7**	2.03517745	3.31298192	12.5752222	0.00130436
**PTGS2**	2.03394061	3.87797019	12.9180506	0.00116569
**NEIL1**	2.03305228	3.24279389	12.2112847	0.00149668
**SLC2A10**	2.02394685	3.38955685	7.47846377	0.01053773
**SH2D5**	2.02132239	3.73237324	5.84087218	0.02249436
**KIF17**	2.01988148	2.35124198	7.21204973	0.01171265
**COL6A6**	2.01321031	3.4510405	7.85673811	0.00892874
**FMO3**	1.99022214	2.88118331	9.08424864	0.00519874
**CFI**	1.98897236	2.52815768	14.4641873	0.00065278
**FGF22**	1.98751261	1.49940038	12.4722927	0.0013559
**PRMT6**	1.98220418	1.46389835	16.2845732	0.00034571
**HLA-C**	1.9816553	9.74765701	7.23148838	0.01222061
**ZNF71**	1.9655158	2.51874163	24.2377449	2.88E-05
**TFPI**	1.95716477	4.47821722	7.96129377	0.00873482
**B3GNT3**	1.95096946	3.35419042	14.7336101	0.00059306
**PEG10**	1.94732746	2.84545952	5.60431149	0.02479185
**S100A12**	1.94266585	6.03582734	5.17360243	0.03123707
**HS3ST4**	1.93881038	2.87405544	4.51444394	0.04246704
**TBL1Y**	1.92631896	2.49284032	7.0157896	0.01275718
**CCL17**	1.92100322	2.58308856	8.48026366	0.00671008
**S100P**	1.91978429	6.33009824	7.26123686	0.01204417
**RAB26**	1.91091206	3.63837824	7.11500781	0.01241817
**ZNF350**	1.90568209	3.28444618	23.3697829	3.71E-05

**ZNF528**	1.89832409	3.58271478	27.7452087	1.09E-05
**HLA-DQA1**	1.89820731	6.18080601	4.99708917	0.03400547
**PLCE1**	1.89656524	2.91307846	7.68283884	0.0095127
**PGBD5**	1.89488319	2.50725751	18.9838931	0.00014162
**ERP27**	1.89285708	1.58636364	13.2297207	0.00102209
**TSPAN1**	1.88905982	6.32344935	5.55096082	0.02611837
**DLX5**	1.88109031	4.03033301	23.6201198	3.45E-05
**SH3GL3**	1.8803638	2.23940503	8.43072635	0.00685363
**L1CAM**	1.86549731	3.42303908	4.45354982	0.04386206
**GNAL**	1.8611325	3.25064034	7.84760047	0.00885698
**LRRK2**	1.85125363	3.35883382	9.73810977	0.00397439
**HIST1H2BF**	1.85013633	1.42039031	13.0302502	0.00110044
**ZG16B**	1.84700974	3.86362378	7.5577692	0.01021445
**SYT8**	1.84695508	3.48938712	9.7890834	0.00389246
**MYO7B**	1.84646264	1.81699645	10.7600277	0.00262825
**CCN5**	1.84405659	3.77296847	5.96036057	0.02117297
**IL17RD**	1.84250221	3.05180327	12.2042643	0.00150068
**ATP13A4**	1.84007533	5.55029405	5.74665158	0.02379098
**ADTRP**	1.8373434	4.29203308	10.1877233	0.00339935
**LGALS2**	1.82566075	3.48182514	7.37469075	0.01100299
**TCHH**	1.81644269	1.73091082	12.6127581	0.00128609
**MROH7**	1.80723295	2.97140039	7.71726478	0.00933965
**THEM5**	1.80606878	2.80570435	5.84486068	0.02203229
**DEGS2**	1.79726184	5.41383436	14.7152323	0.00062343
**ZNF254**	1.78848596	5.26175668	36.4028601	1.26E-06
**OTX1**	1.78540408	3.37569029	13.9711241	0.00077943
**USP9Y**	1.7842536	2.72846716	7.99135081	0.0082826
**SIAE**	1.77749962	4.03530433	31.4136771	4.21E-06
**TOR4A**	1.77419522	4.03987877	22.7322875	4.47E-05
**MROH6**	1.76917682	5.17282045	23.2659169	3.82E-05
**TMEM45B**	1.76326084	4.27150549	23.0154141	4.11E-05
**ADAMTSL5**	1.75781431	3.52664	13.9366114	0.00078923
**USP2**	1.75671714	3.93587397	5.5388482	0.0258448
**RIN1**	1.74160587	4.13249434	12.3723101	0.0014195
**ADAMTS3**	1.73658919	0.98256834	12.0947723	0.00156452
**HES5**	1.73497465	3.87012467	6.86244967	0.01391703
**LAYN**	1.7340034	4.85511785	19.1292974	0.00013521
**VSIG2**	1.72988904	1.66674816	12.6634282	0.00126186
**TMEM238L**	1.72338484	1.98551507	6.0143754	0.02021516
**SCCPDH**	1.71822186	4.28848097	9.49941603	0.00447638
**IGFN1**	1.70959135	2.40888368	6.38392779	0.01701655
**NME4**	1.70310585	5.78493753	27.0368751	1.32E-05

**GPR35**	1.7014511	2.90971175	8.25361107	0.00739468
**FBP1**	1.70100171	5.63789288	5.5519346	0.02605786
**CORIN**	1.69920003	1.45453469	6.30266035	0.01766936
**FMO5**	1.69694612	2.26858505	5.09142942	0.03152976
**EEF1A2**	1.69190336	3.66862879	4.91850271	0.03479881
**SOWAHB**	1.68795177	4.82549556	9.44250721	0.00465347
**THRB**	1.6858261	3.45343549	12.1849658	0.00151172
**FAM86C1**	1.68374183	3.15147457	10.2635777	0.00320473
**ARL14**	1.68204683	3.02647311	6.76805519	0.01429213
**GRIN2D**	1.6785645	3.76359151	14.7433635	0.00059101
**MMP17**	1.6747267	3.7569542	11.2426656	0.00217359
**HS3ST3A1**	1.66976458	5.16588409	10.8381147	0.00264891
**SLC44A3**	1.66271999	2.5103485	14.263662	0.0007014
**STEAP4**	1.6622086	2.25857742	12.5594364	0.00131213
**SCUBE2**	1.65045573	1.98068358	7.31885251	0.0111335
**MISP**	1.64779089	2.22301501	10.2777817	0.00318646
**GPT**	1.64281887	2.65092103	5.04313556	0.03228558
**KCNQ1**	1.64016982	4.04475435	7.9948316	0.00840628
**ZNF681**	1.63572876	3.64569238	19.0496284	0.00013868
**SCNN1A**	1.63459676	5.96549559	9.40099138	0.00476922
**RBP5**	1.63197471	2.03745006	4.33662265	0.04595816
**GABRA3**	1.62718297	2.03525847	10.9156921	0.00247133
**CTF1**	1.62604608	2.3951274	7.64627199	0.00963128
**TFAP2E**	1.62414983	3.31119543	11.8572959	0.00171326
**ZNF888**	1.62118443	3.32558184	16.0173278	0.00037884
**SLC22A18AS**	1.61781157	3.11540165	9.13763621	0.00508406
**PDGFC**	1.61406813	3.76883073	13.4184193	0.00095347
**ZSCAN31**	1.6133759	3.51671937	11.4549287	0.00200112
**TP53INP2**	1.61191292	5.17817805	11.0875505	0.00239031
**FAM171A1**	1.60811835	2.8206115	7.53025474	0.01013678
**PLIN5**	1.60373104	2.13868037	8.75162888	0.00597928
**RHOF**	1.6035058	5.61878405	34.4199088	2.01E-06
**ZNF772**	1.59757432	3.09026462	9.44970242	0.00446608
**SEMA3B**	1.59702277	3.80673554	11.7117427	0.00181189
**PLK2**	1.59474353	5.85170676	10.112306	0.00356865
**PAQR6**	1.58965028	4.10958929	13.8421202	0.00081675
**WNT2**	1.58768388	2.75258519	5.9176359	0.02115719
**RGS17**	1.58583216	1.97231385	7.17485807	0.01187313
**DKK2**	1.58133842	1.77413638	7.15547648	0.01197669
**DOK4**	1.58107021	4.28563212	14.4843965	0.00064808
**HLA-A**	1.57890077	8.26943744	5.32821119	0.02901653
**DENND5B**	1.5736483	3.25548227	15.1939284	0.00050417
**NCCRP1**	1.57239676	6.28262972	8.41209986	0.0072162

**PROSER2**	1.56867894	3.29721993	9.03071752	0.00531654
**KIF26B**	1.56422808	3.97034652	11.6872891	0.00182905
**LRRC15**	1.55523205	4.05311754	5.66471769	0.02427407
**RUNDC3A**	1.55235459	2.58754839	4.78616166	0.03662967
**OPN3**	1.54566513	5.25407989	7.4087627	0.01105304
**EYA4**	1.54099068	1.45850978	6.83939873	0.01381188
**AMN**	1.53895486	4.35797995	13.2859195	0.00100112
**ZFP28**	1.53801344	2.73188252	11.2841601	0.00213865
**MYL12A**	1.53560576	8.26064216	8.29217918	0.00764763
**ZNF426**	1.53546492	6.08756798	11.6920739	0.00190078
**CTH**	1.53316219	2.3540283	10.8654185	0.00252087
**CRACR2B**	1.53029238	4.18412383	7.47467804	0.01054337
**GTF2A2**	1.52527318	3.26331951	13.7044163	0.00085872
**HIST3H2A**	1.52525145	3.27101557	16.8490506	0.0002855
**LRRC20**	1.52414024	3.56025815	13.4302189	0.00094935
**CRISPLD1**	1.52356418	2.45976782	10.2267589	0.00325261
**HSPB8**	1.51870614	5.64210402	5.13791908	0.03161921
**QPRT**	1.51699256	3.16373035	11.3630444	0.0020739
**ICAM4**	1.51287383	1.59240838	5.8424312	0.0219227
**EVA1A**	1.50310159	2.69509092	7.09803358	0.01228944
**MAMDC2**	1.50079108	2.13387925	5.18343452	0.03009245
**CD36**	1.50031819	3.78285186	7.62982759	0.00973884
**SYTL5**	1.50002455	1.59304375	4.64106052	0.03935828
**KCNG1**	-1.5036064	1.7657366	14.3899413	0.00067034
**ACACB**	-1.5040017	3.14948987	29.9166978	6.16E-06
**SPDYE5**	-1.5046396	1.39302057	5.7410233	0.02300334
**GPC2**	-1.5096166	3.83466009	13.1444864	0.00105481
**LOX**	-1.5200143	4.7689394	9.55579225	0.00436901
**CAMK2N2**	-1.5207377	4.272262	4.65701185	0.0397413
**MYLK2**	-1.5295621	1.30079601	8.2753158	0.00732596
**GBP6**	-1.5368945	7.41415045	57.1067286	1.98E-08
**GAD1**	-1.5378463	2.26472613	5.18817835	0.03002285
**EGF**	-1.5452133	1.66422775	6.00237568	0.02032948
**NR4A1**	-1.5700989	6.17854245	8.83931429	0.00601367
**GLI1**	-1.5732311	1.29126979	8.12837876	0.00780509
**PKDCC**	-1.5743685	2.79131605	10.7700077	0.00261787
**RYR3**	-1.5769954	1.57138024	5.31409905	0.02823848
**SYNPO2**	-1.5835087	3.68534892	20.1575077	9.77E-05
**TRPM6**	-1.5969594	1.23041175	9.8248201	0.00382849
**FOXD4**	-1.6035835	1.09165232	10.2070668	0.00327853
**GNAZ**	-1.6140912	1.64484728	8.08084578	0.00796726
**EXOC3L4**	-1.6162811	3.41839206	15.7541766	0.00041481

**SCN9A**	-1.6220447	3.18009194	4.55137464	0.04148171
**NHLH2**	-1.6313509	2.19866822	8.68406307	0.00615282
**RRM2**	-1.6344471	7.4191426	14.2415752	0.00076139
**HLF**	-1.6368145	2.08423029	13.9209157	0.00079373
**B4GALNT1**	-1.6544737	3.04702889	6.13884174	0.01913075
**SLC16A11**	-1.7055111	2.11514439	9.91815893	0.00368558
**LYPD6**	-1.7074233	3.1535853	9.41013506	0.00453974
**RADX**	-1.7262339	2.99245189	6.26115045	0.01809852
**SCIN**	-1.7356025	2.75979492	11.6532907	0.0018532
**CPA6**	-1.7408457	2.8302914	4.54200939	0.04167717
**IGFL3**	-1.7422709	2.47243568	7.71901831	0.0093283
**IFIT2**	-1.7581896	4.22999958	4.72918339	0.03847076
**COX6B2**	-1.7631346	4.04972322	10.8275386	0.00259044
**CADM3**	-1.7639869	2.09312553	5.35512695	0.02775405
**GLS2**	-1.8379264	2.42762771	10.3775119	0.00306132
**BCO1**	-1.8479928	1.50944643	12.0353802	0.00160037
**SEPTIN3**	-1.8485704	2.09768939	8.74898177	0.00598598
**PRIMA1**	-1.8529975	3.65893212	13.381753	0.00096641
**PRLR**	-1.8579711	1.69795017	8.03281003	0.00813487
**KCNA2**	-1.9006341	2.34990076	7.10733984	0.01226131
**COL22A1**	-1.9107407	2.31926253	5.76143333	0.02291296
**MYO3A**	-1.9661985	2.60207343	6.37563258	0.017215
**MSMB**	-2.0135553	3.61502211	12.6146016	0.00129097
**HSPA6**	-2.0367768	3.4670635	14.1005087	0.00074382
**SFTPD**	-2.0436867	1.92152081	12.7706288	0.00121221
**EBF4**	-2.0473552	4.29357901	34.8717941	1.81E-06
**TCERG1L**	-2.0475316	2.19643421	14.2321011	0.0007094
**CRMP1**	-2.0622978	3.51922223	6.56944959	0.01600842
**TMTC1**	-2.0771686	2.3616753	7.59921534	0.00987489
**MYBPC1**	-2.1086959	2.36830406	6.94596256	0.01326675
**SLC6A17**	-2.1218319	2.61774916	7.94477319	0.00851494
**KCNS3**	-2.12747	5.65717597	16.9311497	0.00030548
**A2ML1**	-2.1437653	4.52016719	10.1284607	0.00357495
**Dec-01**	-2.1517632	2.17476041	8.94044019	0.0055263
**GLB1L2**	-2.1624031	1.87295518	10.749615	0.00263912
**SOX30**	-2.1835995	2.00511488	9.8813374	0.00374126
**SHCBP1L**	-2.2129738	3.89152412	6.15411035	0.01953423
**ABCA3**	-2.2459672	3.58885433	12.0951177	0.00160421
**FAM107A**	-2.2954505	2.68259812	9.88600083	0.00375699
**PHACTR3**	-2.298919	3.04185058	7.03520757	0.01289946
**PLA2G2A**	-2.3227891	2.98511841	9.85616177	0.00383544
**C2CD4C**	-2.4168633	1.76251626	11.5273869	0.00194567
**TNFSF18**	-2.4741364	1.64318372	20.3793721	9.12E-05

**SULT1E1**	-2.4969033	2.40563629	9.86990178	0.00379763
**IGHV5-10-1**	-2.5007221	1.60315547	9.93992283	0.0036533
**MYEOV**	-2.5164449	5.83669245	8.40028179	0.0074318
**IRX6**	-2.522332	2.90933772	10.370723	0.00313861
**ARNT2**	-2.5515129	3.10524991	17.0947515	0.00026289
**OLFM1**	-2.6117724	6.27806166	23.0810751	4.77E-05
**ATP12A**	-2.748	3.69257691	9.57940115	0.00446393
**GABRP**	-2.8541708	6.21113179	15.1190323	0.00060283
**STAG3**	-2.9618939	4.68326501	15.422519	0.0005241
**CYP4B1**	-2.9656805	3.02738575	13.2624944	0.00104549
**TPO**	-3.0277791	2.66730107	16.0413659	0.0003804

Table S4. Semi-quantitative pathologist scores for immunohistochemical expression of candidate genes.

Condition	Sample number	PNPLA3	MARCO	PAEP	Patient
Recurrence	ctc-044-28310	1	1	0	R3
Recurrence	ctc-037-9237	3	3	0	R2
Recurrence	ctc-040-18588	3	2	1	R1
Recurrence	603922 ctc-48	1	0	0	R4
Non-recurrence	ctc-001-09900	0	0	0	NR1
Non-recurrence	ctc-008-18262	1	0	0	NR3
Non-recurrence	ctc-012-28724	2	0	0	NR4
Non-recurrence	ctc-027-52393-8	0	0	0	NR5
Non-recurrence	ctc-041-25115	1	2	0	NR2
Non-recurrence	35384	3	2	0	NR6

Table S5. Effect size and p-value of linear mixed-effects model for candidate genes

Gene	effect size	p-value
PAEP	0.1070734	0.04825094
PNPLA3	0.06383171	0.07380583
FMO2	0.073839351	0.1362927
MARCO	0.029921516	0.1738644
